# The body temperature of *Tyrannosaurus rex*

**DOI:** 10.1126/sciadv.aeb7653

**Published:** 2026-09-16

**Authors:** Randon J. Flores, Robert A. Eagle, Robin B. Trayler, Gabriele Larocca Conte, Sora L. Kim, Alfio Alessandro Chiarenza, Alex Farnsworth, Paul J. Valdes, Luis Chiappe, Aradhna Tripati

**Affiliations:** ^1^ Department of Earth, Planetary, and Space Sciences, University of California–Los Angeles, Los Angeles, CA, USA.; ^2^ Institute of the Environment and Sustainability, University of California–Los Angeles, Los Angeles, CA, USA.; ^3^ Department of Atmospheric and Oceanic Sciences, University of California–Los Angeles, Los Angeles, CA, USA.; ^4^ School of Geographical Sciences and Cabot Institute for the Environment, University of Bristol, Bristol, UK.; ^5^ School of Earth Sciences, University of Bristol, Bristol, UK.; ^6^ Department of Life and Environmental Sciences, University of California–Merced, Merced, CA, USA.; ^7^ Schmid College of Science and Technology, Chapman University, Orange, CA, USA.; ^8^ Department of Earth Sciences, University College London, Gower Street, London WC1E 6BT, UK.; ^9^ North Carolina Museum of Natural Sciences, Raleigh, NC, USA.; ^10^ State Key Laboratory of Tibetan Plateau Earth System, Environment and Resources (TPESER), Institute of Tibetan Plateau Research, Chinese Academy of Sciences, Beijing, China.; ^11^ Dinosaur Institute, Natural History Museum of Los Angeles County, Los Angeles, CA, USA.

## Abstract

Despite an extensive history of study, quantitative constraints on the thermophysiology of *Tyrannosaurus rex* remain limited. Here, we derive a thermodynamically based determination of *T. rex* body temperatures from clumped isotopes in tooth enamel. Measurements of three *T. rex* teeth from the Late Cretaceous Hell Creek Formation yield a temperature of 36.3 ± 2.5°C (1 SE), comparable to modern endotherms. Teeth of coeval Hell Creek Formation crocodilians yielded temperatures significantly lower than did those of *T. rex.* Comparison with proxy-derived paleotemperatures and model simulations indicates that *T. rex* maintained a higher body temperature than its environment. Projected habitat suitability for *T. rex* based on these results shows that most of the North American paleocontinent, including colder and higher latitudes, were potentially accessible to the species.

## INTRODUCTION

Thermophysiology plays a crucial role in determining animal behavior and ecology (*1*). Paleophysiological studies are therefore essential for understanding how non-avian dinosaurs (hereafter referred to as “dinosaurs”) evolved and interacted with the varied climates and environments of the Mesozoic. While a consensus on thermophysiology across the clade Dinosauria has yet to be reached, current evidence from anatomy (*2*), bone histology (*3*), energetics modeling (*4*), phylogenetics, and geographic distribution (*5*, *6*) suggests that endothermy developed in at least some dinosaur groups. Endotherm-like body temperatures have also been identified for select genera within Theropoda, Sauropoda, and Ornithischia through oxygen isotope paleothermometry (*7*, *8*) and clumped isotope paleothermometry (*9*–*13*), suggesting that elevated body temperatures, and therefore high metabolic rates, were prevalent among dinosaurs. However, recent evidence from fossil biomolecules (*14*) as well as macroevolutionary modeling and ancestral state reconstruction of paleoclimatic niches (*5*) is consistent with the view that not all dinosaur clades maintained endothermic metabolisms and instead used a range of thermophysiological strategies in accordance with their environments and ecology (*4*). This apparent complexity lends to the continued scientific debate on physiology and thermal regulation in specific dinosaur taxa.

*Tyrannosaurus rex* is perhaps the most intensely studied dinosaur owing to a relatively well-sampled record (*15*), earning it the status of an exemplar organism that is commonly referenced in studies of vertebrate paleontology (*16*). Aspects of the biology and ecology of *T. rex*, which provide evidence for an endothermic thermophysiology, include locomotor costs (*17*), growth rates (*18*), and geographic distribution (*5*), yet confident estimates of body temperature are lacking. Prior isotopic works applied to *T. rex* bone phosphate (*19*) and tyrannosaurid tooth enamel (*7*, *8*) are limited due to the confounding effect of body water composition, which is difficult to constrain.

Carbonate clumped isotope paleothermometry provides an alternative approach for determining body temperature independent of body water composition through the direct determination of mineral formation temperatures based on the abundance of ^13^C-^18^O bonds in carbonate minerals (*20*). Clumped isotopes in bioapatite minerals (e.g., enamel, dentin, and bone) reflect vertebrate body temperatures (*10*), allowing for the determination of body temperatures in extinct taxa (*10*, *21*–*23*). Here, we calculated *T. rex* body temperatures using clumped isotope thermometry and compared these values to those derived from contemporaneous ectotherms. We then compared *T. rex* and crocodilian body temperatures to published clumped isotope-derived environmental temperatures to identify offsets that could indicate differing thermophysiological strategies. Last, we used estimated body temperature as a basis to model thermophysiological response to the paleoenvironment and habitat suitability for *T. rex* under simulated Late Cretaceous climate simulations.

Three *T. rex* teeth were provided for analysis by the Natural History Museum of Los Angeles County (LACM) including two teeth associated with specimen LACM 150167 (LACM 150167 EEEE3 and LACM 150167 PPPP) and one isolated partial *T. rex* tooth (LACM 151468). Specimens were collected from the Late Cretaceous Hell Creek Formation (HCF) from a site southeast of Ekalaka, Carter County, Montana. LACM 150167, nicknamed Thomas, is a well-preserved young adult *T. rex* specimen that would have likely exceeded 3000 kg in life (*24*). Additionally, five crocodilian teeth from the HCF were provided by the Raymond M. Alf Museum of Paleontology (RAM). These teeth were collected from the upper Maastrichtian vertebrate fossil site UCMP V85092 (RAM site V--1994084) near McGuire Creek in McCone County, Montana.

## RESULTS

### Sample preservation

Determining the preservation of primary isotopic composition is essential in isotope thermometry. While there is no singular diagnostic method for ensuring original isotopic composition in fossil specimens, we can assemble information from diverse approaches. All body temperature estimates in this study are based on measurements of enamel, which is known to have the highest potential for preserving original isotopic signatures at geologic timescales compared to other skeletal apatites (*25*). To establish the state of enamel preservation, we conducted multiple independent evaluations of diagenesis in fossil teeth and found no clear signs of post-burial alteration.

First, we observed distinct isotopic values in HCF teeth where diagenesis would be expected to similarly affect isotope compositions of fossils recovered from the same strata (*25*). To identify preservation of isotope values consistent with the expected ecology of *T. rex* and crocodilians, we compared δ^13^C of teeth measured in this study with δ^13^C of hadrosaurids, ceratopsians, and gar reported by Fricke *et al*. (*26*) (fig. S3). Our results show agreement with dietary offsets observed in another Late Cretaceous fossil assemblage reported in (*27*) where both tyrannosaurids and crocodilians have lower δ^13^C than their respective prey. The distinct clumped isotope-derived temperatures for *T. rex* and crocodilians similarly suggest that homogenous diagenetic overprinting of original isotopic composition is unlikely for these specimens. The difference in HCF *T. rex* and crocodilian body temperatures is consistent with clumped isotope-derived temperatures of modern large mammals and crocodilians (*28*), suggesting preservation of realistic biological signals.

Second, we compared the carbon and oxygen isotope composition of the enamel and dentin components of teeth from LACM 150167 and LACM 151468 given the assumption that these structural components would yield discrete isotope values as a result of different preservation potential of enamel and dentin (*25*). Enamel and dentin of HCF specimens have distinct isotopic compositions (fig. S2), consistent with diagenetic alteration of the less coarsely crystalline dentin but not necessarily enamel.

Third, we compared the carbonate and phosphate δ^18^O values measured from the same teeth to identify diagenetic effects on one moiety relative to another (*29*–*31*) (fig. S1). Measurements of *T. rex* teeth in this study yielded offsets ranging from 9.8‰ to 10.4‰, consistent with the overall range of 6.6‰ to 10.6‰ observed in modern mammals (*29*, *32*). Measurements of crocodilian teeth range from 8.4‰ to 11.7‰. While the 11.7‰ value obtained from 2 crocodilian teeth is higher than the ∼9‰ value typically considered indicative of well-preserved samples, concerns have been raised regarding the reliability of this approach as a diagenetic screening parameter due to lacking constraints on how factors including diet, seasonality, physiology, and body temperature influence PO_4_-CO_3_ δ^18^O spacing (*32*–*34*) (see Supplementary Text for more details). For these reasons, we do not interpret this range as clear evidence for alteration in crocodilian teeth.

Last, we used Fourier transform infrared (FTIR) analyses to identify potential chemical alteration in fossil bioapatite. FTIR analyses show that measured HCF tooth specimens are more similar to modern alligator teeth than older Jurassic dinosaur teeth, and are dissimilar to a previously reported chemically altered diagenetic endmember (*10*). Additionally, the carbonate contents of specimens determined through FTIR measurements range from 6.3 to 7.4 wt %. This is in close agreement with values calculated for modern reptilian enamel (*35*), suggesting that there was no addition of post-burial exogenous inorganic carbonate in these specimens.

### *T. rex* and crocodilian body temperatures

**Fig. 1.**
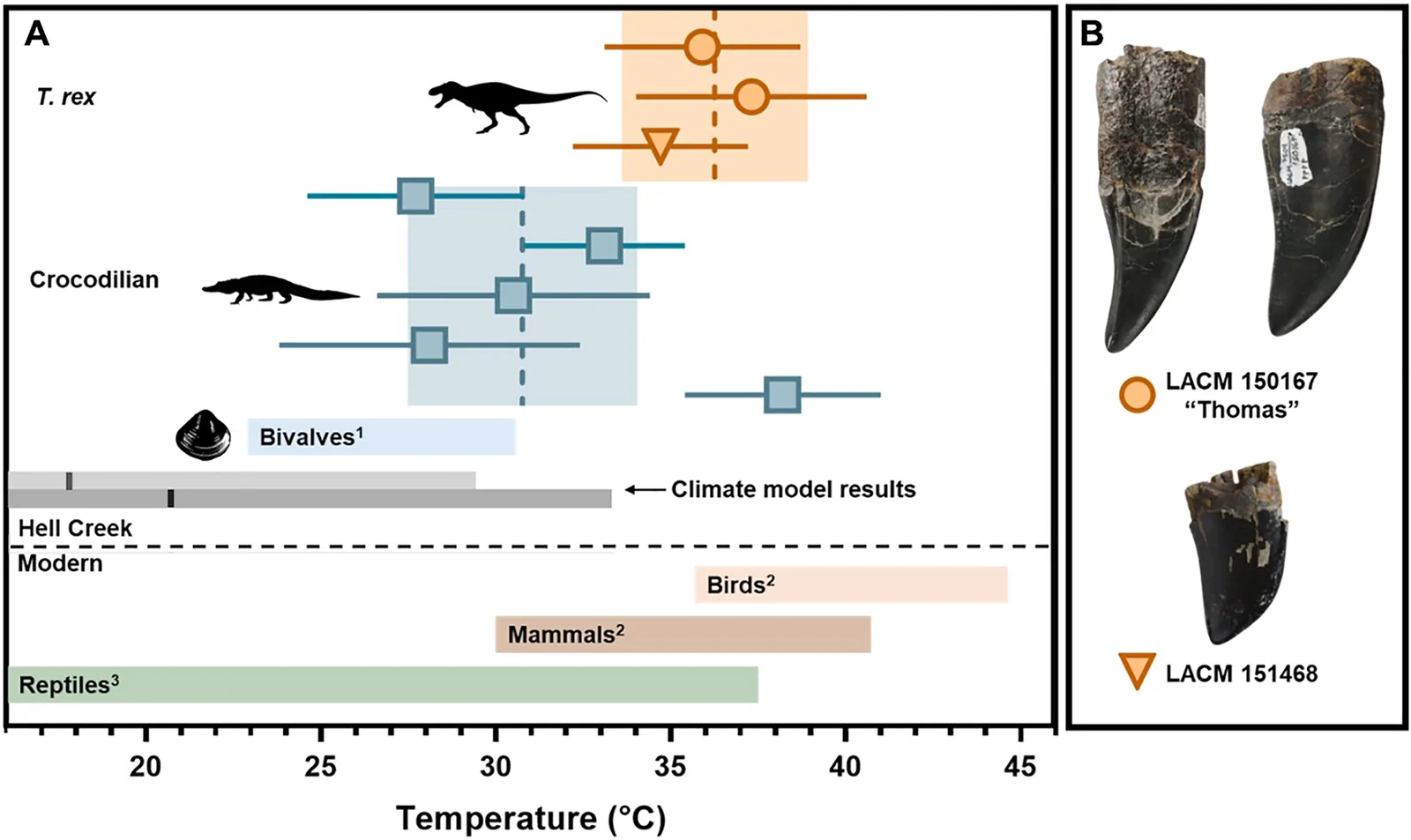
Comparison of calculated Δ_47_ temperatures for *T. rex*, crocodilians, and bivalves from the HCF along with body temperature ranges of modern birds, mammals, and reptiles. (**A**) Symbols indicate sample averages for individual tooth specimens, with error bars indicating 1 SE. For *T. rex*, circles indicate samples from LACM 150167 teeth, and the triangle indicates the single LACM 151468 tooth. Vertical dashed lines indicate taxon averages, and shaded regions under data points indicate 95% confidence interval (CI). Bivalve and modern animal bars indicate the full range of temperatures reported in their respective studies: The upper light gray bars indicate model results and show yearly temperature range at 3× CO_2_ concentration, while the lower dark gray bar shows 6× CO_2_ concentration. Both bars show the full range of monthly temperatures with a dark vertical line to indicate mean annual temperature. ^1^Tobin *et al*. (*37*), ^2^Clarke and Rotherty (*38*), ^3^Eagle *et al*. (*11*). All silhouettes are licensed under Creative Commons license CC0 1.0 (https://creativecommons.org/publicdomain/zero/1.0/). Artist credits: Manuel Brea Lueiro (*T. rex*), Guillaume Dera (crocodilian), Pablo Amador Crespo (bivalve). (**B**) Photographs of *T. rex* tooth specimens used in this study (photo credit: S. Abramowicz, Dinosaur Institute, Natural History Museum of Los Angeles County).

Clumped isotope-derived temperatures (Table 1 and Fig. 1) from tooth enamel of the two LACM 150167 teeth yielded temperatures of 37.3 ± 3.3°C (1 SE) and 35.9 ± 2.8°C for LACM 150167 PPPP and LACM 150167 EEEE 3, respectively, and an average temperature of 36.6 ± 2.8°C (1 SE) for both teeth. The second *T. rex* specimen, LACM 151468, yielded a temperature of (34.7 ± 2.5°C), resulting in an average body temperature of 36.3 ± 2.5°C (1 SE) based on all three teeth. Temperatures for individual crocodilian teeth range from 27.7° to 38.2°C and produce an overall average value of 30.9 ± 2.6°C (1 SE) for all tooth measurements (Table 1 and Fig. 1). Estimated body temperatures for crocodilians and *T. rex* are significantly different [Welch’s *t* test, *t*(22.99) = 2.78, *P* = 0.0108].

**Table 1.** Isotopic results for *T. rex* and crocodilian tooth data. *n* = number of clumped isotope measurements.

Sample	*n*	δ^13^C VPDB	1 SD	δ^18^O_c_ VSMOW	1 SD	Δ_47_	1 SE	TΔ_47_ °C*	1 SE	δ^18^O_bw_ VSMOW†	1 SD	δ^18^O_env_ VSMOW‡
LACM 150167 PPPP	5	−8.3	0.1	20.9	0.2	0.560	0.006	37.3	3.3	−7.5	1.0	−13.7
LACM 150167 EEEE 3	6	−8.8	0.2	19.8	0.1	0.564	0.005	35.9	2.8	−8.8	1.0	−12.5
LACM 151468	2	−6.0	0.1	20.8	0.2	0.567	0.004	34.7	2.5	−8.1	0.5	−11.9
Thomas teeth avg	11	−8.6	0.3	20.3	0.6	0.562	0.004	36.6	2.7	−8.2	1.2	−13.1
*T. rex* teeth avg	13	−8.2	1.0	20.4	0.6	0.563	0.003	36.3	2.5	−8.2	1.1	−12.7
HCF Croc 1	3	−7.7	0.2	18.7	0.5	0.586	0.006	27.7	3.1	−11.4	0.7	−13.4
HCF Croc 2	3	−6.3	0.1	19.4	0.1	0.585	0.011	28.1	4.3	−10.7	1.3	−12.9
HCF Croc 3	3	−6.8	0	20.8	0.3	0.578	0.009	30.5	3.9	−8.8	1.3	−10.3
HCF Croc 4	2	−7.3	0.1	22.1	0.2	0.558	0.005	38.2	2.8	−6.2	0.6	−7.9
HCF Croc 5	2	−6.5	0.1	19.3	0	0.571	0.002	33.1	2.3	−9.9	0.2	−11.5
Crocodilian teeth avg	13	−6.9	0.6	19.9	1.2	0.577	0.004	30.9	2.6	−9.6	2.0	−11.1

* Temperatures calculated using the equation of Anderson *et al*. (*60*) where errors in Δ47 measurement and calibration uncertainties are propagated.

†Body water calculated from TΔ_47_ and δ^18^O_c_ using the apatite carbonate-oxygen isotope temperature fractionation equation of Löffler *et al*. (*23*).

‡Environmental water calculated from phosphate δ^18^O and phosphate-water isotope fractionation equations of Amiot *et al*. (*48*) for *T. rex* and Amiot *et al*. (*47*) for crocodilians.

### Comparison with environmental temperatures

#### Model temperatures

Realistic late Maastrichtian numerical simulations were produced using a new high-resolution atmosphere-only (∼60-km gridbox) paleoclimate model at both 3× and 6× pre-industrial CO_2_ levels (840 and 1680 ppmv, respectively) with the Robertson paleogeography (fig. S5, see Supplementary Text for more details) and a 70-million-year (Ma) solar constant. Sea-surface temperatures (SSTs) and sea-ice boundary conditions were driven from the equivalent low-resolution coupled atmosphere-ocean version (∼300-km grid box) of the paleoclimate model, where the only difference is the higher spatial model resolution and more detailed paleogeography. Critically, this version of the model captures polar warmth in line with proxy evidence (*36*). Simulated air temperatures averaged over the region of the HCF relevant to this study (paleolatitude 49° to 53°N, 75° to 85°W) at 3× CO_2_ yielded warm month temperatures of 24.9° to 29.5°C and mean annual temperatures of 17.8°C. Simulated air temperatures at 6× CO_2_ yielded warm summer month temperatures of 27.7° to 33.4°C and mean annual temperatures of 20.7°C (fig. S6).

#### Bivalves

Environmental temperatures for the HCF over the last ∼300 thousand years (ka) of the Maastrichtian were reported in (*37*) based on clumped isotope measurements of unionid bivalves. Temperatures over this interval range from 22.6° to 30.5°C and yield an average of 25.9 ± 1.2°C (1 SE) (Fig. 1). Modern unionid bivalve Δ_47_ values reflect water temperatures in agreement with summer air temperatures, suggesting that HCF bivalves likely preserve warm season environmental temperatures (*37*). Our estimated *T. rex* body temperatures are significantly warmer than bivalve-derived temperatures (Mann-Whitney test, *z* = −4.25, *P* ≤ 0.0001).

## DISCUSSION

### Body temperature in *T. rex* differs from environment and contemporaneous ectotherms

Body temperature is not an unambiguous indicator of physiology and metabolic rate; however, the estimates provided here for *T. rex* reveal important insights with relevance to its ecology and biology and add to a growing body of evidence for endothermy in theropod dinosaurs. The clumped isotope-derived *T. rex* body temperature of 36.3 ± 2.5°C is in agreement with those of extant large mammals such as Indian and African elephants with reported body temperatures of 36.0° and 36.2°C, respectively (*38*). This temperature is also within uncertainty of large flightless birds such as extant ostriches and emus, which exhibit body temperatures ranging from 38.1° to 39.7°C (*39*) and 37.7° to 38.3°C (*38*), respectively, but slightly lower than smaller flighted birds with an average body temperature of 41.6 ± 0.5°C (1 SE) (*38*). *T. rex* body temperatures are warmer than those of coeval bivalves and simulated air temperatures at both CO_2_ concentrations, indicating that *T. rex* maintained a body temperature higher than its environment. While in the case of *T. rex* specimens, teeth were sampled from a limited part of the tooth crown (with the intention of minimizing damage to teeth), which could potentially introduce the possibility of seasonal growth bias, we found that the samples taken from two different areas of two separate teeth from LACM 150167 yielded statistically indistinguishable temperature estimates (Welch’s *t* test *P* = 0.66). While it is impossible to conclusively rule out a seasonal bias given the limited small sample size and limitations on microsampling imposed by the material requirements of clumped isotope measurements, the three teeth analyzed show no clear outliers in temperature that would indicate that a seasonal temperature bias is present.

*T. rex* could have also maintained a higher body temperature than contemporaneous ectotherms with behavioral thermoregulation as evidenced by the significant difference in temperature between *T. rex* and crocodilians. Crocodilians regulate their body temperature by moving between water and basking spots on land, resulting in body temperatures up to ∼5°C higher than ambient temperatures (*40*, *41*). Modern crocodilians inhabit a preferred temperature range of ∼30° to 35°C (*42*) with a critical temperature of 38° to 39°C beyond which death may occur (*43*). Our HCF average crocodilian body temperatures are consistent with this range (Table 1) even when considering the specimen that yielded the warmest temperature of 38.2 ± 2.8°C. This particular specimen yields a high temperature but is within 2 SE of the preferred temperature range, illustrating the importance of considering the error in measurements and the additional power from analyzing multiple samples. Data taken across all specimens suggests that the clumped isotope measurements reflect realistic body temperatures for crocodilians. The significant difference in *T. rex* and crocodilian body temperatures could therefore indicate differences in thermophysiology. However, the possibility that large dinosaurs were able to attain high stable body temperatures despite maintaining low metabolic rates through inertial homeothermy (*4*, *44*) must also be considered. While the average temperature from LACM 150167 is higher than the predicted temperature for an ectotherm of equivalent size based on the body size scaling relationships of (*4*, *44*), this model is debated (*45*) and caution must be taken in basing inferences of metabolic rate on body temperature alone.

### Physiological controls on *T. rex* and crocodilian body water

Vertebrate body water δ^18^O (δ^18^O_bw_) is known to be offset from the composition of ingested sources of water as a result of ecology and physiology (*46*). It is therefore possible to make inferences on the ecology and physiology of an animal if the δ^18^O of body water and ingested water are constrained. To identify this offset in *T. rex* and HCF crocodilians, we first constrained δ^18^O_bw_ in all fossil specimens by applying the structurally bound carbonate-water δ^18^O fractionation equation of Löffler *et al*. (*23*) (Table 1). Ingested water (e.g., drinking and food) is derived from environmental water (e.g., meteoric and surface; δ^18^O_env_) (*46*); thus, environmental water can be used as a proxy for ingested water composition when in situ measurements of food and drinking water are not directly available (*47*). To constrain δ^18^O_env_ values in HCF crocodilians, we applied the phosphate-water isotope fractionation equation of Amiot *et al*. (*47*) determined for extant crocodilians, and for *T. rex*, we applied the phosphate-water isotope fractionation equation of Amiot *et al*. (*48*) determined for extant birds. While the bird phosphate-water isotope fractionation equation may not represent a perfect model for reconstructing δ^18^O_env_ from *T. rex* tooth phosphate, Alvarez *et al*. (*49*) found that this equation yielded δ^18^O_env_ values from theropod teeth, consistent with those obtained from Late Cretaceous crocodilians calculated using the equation of Amiot *et al*. (*47*). This agreement is also broadly reflected in our estimates of δ^18^O_env_ values for HCF specimens (Table 1), suggesting that the bird phosphate equation of Amiot *et al*. (*48*) can be a reliable means of reconstructing δ^18^O_env_ when applied to theropod dinosaurs.

**Fig. 2.**
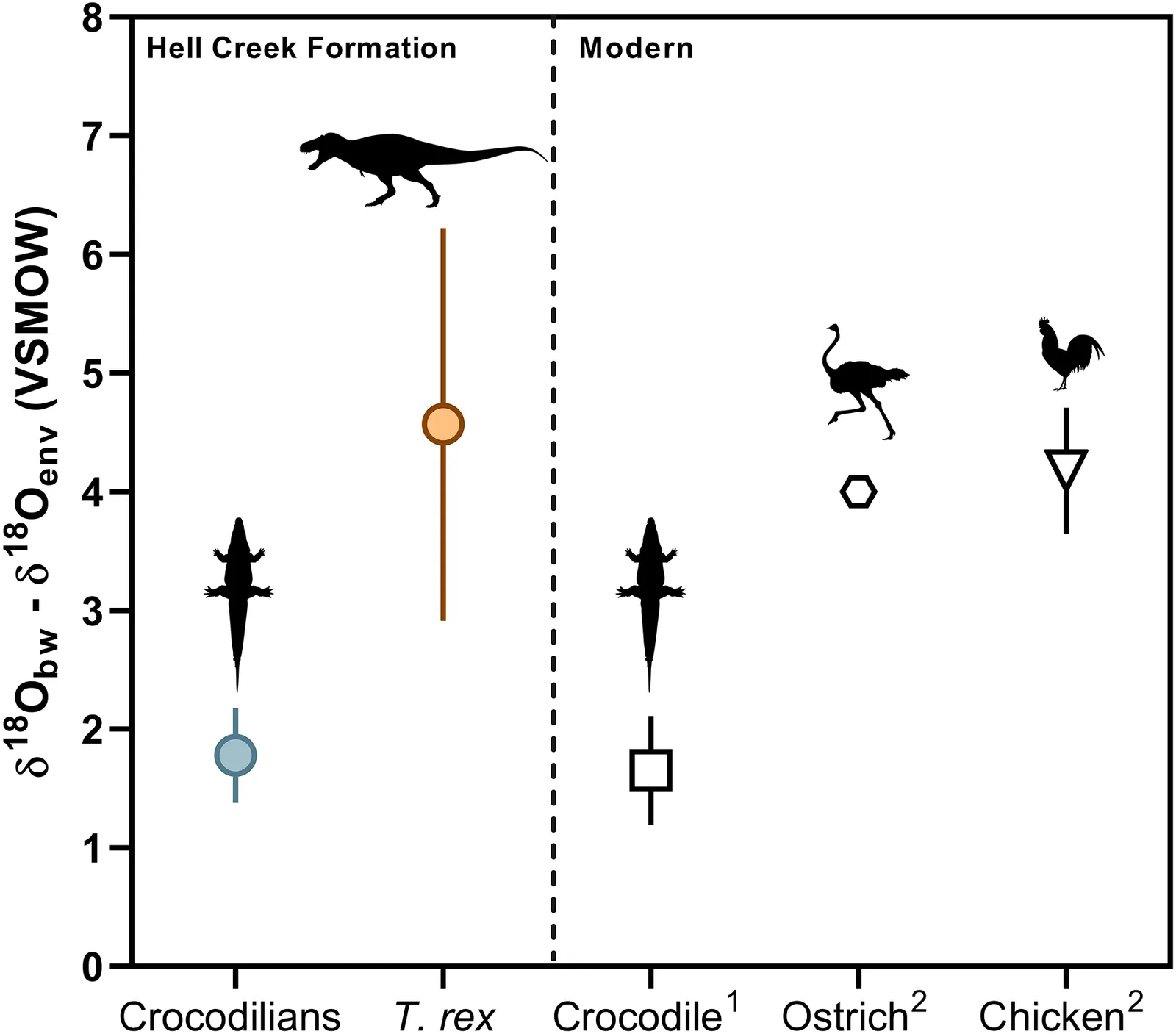
Comparison of calculated environmental and body water δ^18^O in *T. rex* and crocodilians from the HCF with modern crocodiles (*Crocodylus niloticus*), ostrich (*S. camelus*), and chicken (*G. gallus*). Symbols represent the mean offset between measurement-based estimates of body water and environmental δ^18^O, and error bars indicate 95% CI. The ostrich lacks error bars as it is represented by a single data point. ^1^Amiot *et al*. (*47*), ^2^Lazzerini *et al*. (*50*). All silhouettes are licensed under Creative Commons license CC0 1.0 (https://creativecommons.org/publicdomain/zero/1.0/). Artist credits: Manuel Brea Lueiro (*T. rex*), Guillaume Dera (crocodilian), Ferran Sayol (ostrich), Steven Traver (chicken).

Comparison of HCF crocodilian δ^18^O_bw_ with reconstructed δ^18^O_env_ results in an offset of 1.8 ± 0.3‰ (1 SD), consistent with the ∼2‰ offset between body water and environmental water δ^18^O observed in modern crocodilians (*47*) (Fig. 2). Comparing *T. rex* δ^18^O_bw_ with reconstructed δ^18^O_env_ results in an average offset of 4.5 ± 0.7‰ (1 SD), which is similar to mean body water-drinking water offsets in extant birds, including ostrich *Struthio camelus* [4.0‰, (*50*)] and chicken *Gallus gallus* [4.2 ± 1.6‰, 1 SD, (*50*)]. These results are consistent with expected biologies of *T. rex* and crocodilians and provide evidence that the difference in physiological controls on body water composition in these taxa is comparable to differences between modern terrestrial endotherms and semi-aquatic ectotherms.

### Thermal tolerance allows broad geographic range for *T. rex*

While it is impossible to conclusively constrain thermoregulatory strategy from body temperature alone, several lines of evidence point to homeothermy and possibly tachymetabolic endothermy in *T. rex*. Young juvenile tyrannosaurid remains from Maastrichtian deposits of the North Slope of Alaska (∼85° paleolatitude); Druckenmiller *et al*. (*51*) suggest year-round persistence at paleolatitudes, which would have seen seasonal temperatures below freezing (fig. S6). The year-round persistence of large-bodied tyrannosaurids at high paleolatitudes implies physiological cold tolerance compatible with homeothermic endothermy. Macroevolutionary evidence for cold tolerance within the tyrannosaur lineage comparable to that of modern birds supports this inference (*5*, *14*). The elevated endotherm-like body temperatures determined from our isotopic analyses align with these observations, adding to the body of evidence in support of *T. rex* as a homeothermic endotherm.

**Fig. 3.**
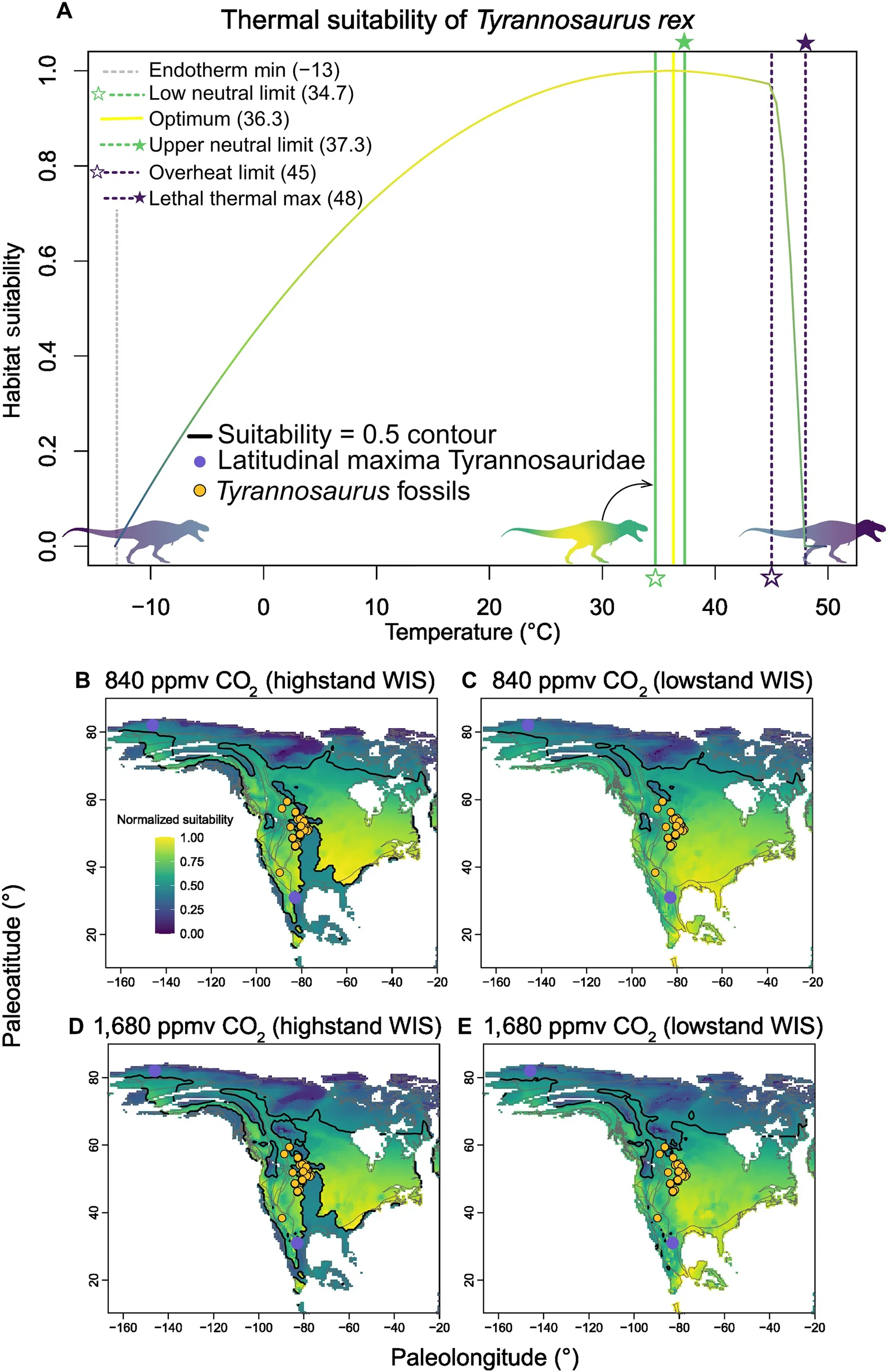
Modeled thermal suitability for *T. rex* based on neutral thermal range inferred from isotopic evidence and on modern endotherm thermal limits. Modelled thermal suitability curve for *Tyrannosaurus rex* (**A**). Modern endotherm thermal limits are derived from (*52*) (see text). Orange points represent *Tyrannosaurus* occurrences, while violet dots represent latitudinal outliers for Tyrannosauridae (see text). Climatically suitable areas on North American paleogeographies (**B**) range from highest suitability at a value of 1 in light green to unsuitable areas at a value of 0 in dark blue. Paleoclimate models show two different CO_2_ emission scenarios [840 ppmv in (B) and (**C**) and 1680 ppmv in (**D**) and (**E**)], while (B) and (D) show the highstand of the Western Interior Seaway (WIS) for the late Maastrichtian, with absolute lowstand in (D) and (E).

With the evidence for endothermy in *T. rex* taken into consideration, we created a physiological response curve for *T. rex*’s thermal tolerance range based on thermal limits observed in extant homeothermic endotherms (Fig. 3A, see Supplementary Text for more details). We reconstructed a thermal range between 34.7° and 37.3°C based on our isotopic results as the peak set point for thermal suitability, gradually moving toward the thermal extremes of −13°C and 43.6°C as documented in modern endotherms (*52*). The upper-temperature tail of the modeled thermal suitability curve smoothly declines from the thermoneutral zone toward temperatures of approximately 45° to 48°C. These temperatures closely approach the lethal thermal limits represented by wet-bulb temperatures (fig. S7) above 35°C, providing a plausible upper boundary for overheating stress in living endotherms (*53*).

Applying the physiological response curve to seasonal temperature variables, along with a relaxed logistic response curve for seasonal precipitation variables, we adopted a mechanistic virtual species approach to model *T. rex* fundamental ecological niche and project it onto North American paleogeography (Fig. 3, B to E). Most fossil occurrences of *T. rex* align well with areas of high modeled habitat suitability across late Maastrichtian North America; however, these known localities represent only a fraction of the total predicted suitable habitats available across the paleocontinent due to spatial biases in their fossil record (*54*). While precise overheating thresholds for *T. rex* remain uncertain, the consistently high suitability predicted across mid-latitudes for both simulated CO_2_ emission scenarios aligns with extensive low-latitude range occupation by this taxon. Furthermore, relatively high modeled suitability values extending into extreme latitudes corroborate latitudinal outlier occurrences for Maastrichtian tyrannosaurids, including the Kikak-Tegoseak Quarry in Alaska’s North Slope (*55*) and cf. *Tyrannosaurus* from Trans Pecos, Texas (*56*), implying a broad latitudinal range for *T. rex*. The ability for *T. rex* to inhabit cooler, high latitudes could also align with recent evidence for a Maastrichtian dispersal of the *Tyrannosaurus* line from Asia across the Bering Land Bridge (*57*).

## MATERIALS AND METHODS

### Isotopic analysis

To perform clumped isotope analyses, enamel and dentin were sampled from tooth specimens using a low-speed rotary drill with a tungsten carbide tipped drill bit and subjected to a pretreatment process consisting of a 1-hour wash in 1 M acetic acid buffered to pH 5 (*13*) and a 4-hour wash in 3% H_2_O_2_. Samples were dried overnight in a 50°C oven following treatment. Clumped isotope measurements were performed in the Tripati-Eagle Lab at the University of California, Los Angeles from December 2023 through November 2024 on a Nu Perspective Isotope Ratio Mass Spectrometer (Nu Instruments-AMETEK, UK) with a custom common acid bath sample preparation apparatus.

Samples were digested in a common acid bath containing 105 wt % phosphoric acid reacted at 90°C followed by multiple steps of cryogenic purification and passage through an adsorption trap packed with Porapak Type-QTM 50/80. Following sample purification, CO_2_ gas was then introduced into the isotope ratio mass spectrometer (IRMS) system via an automated changeover block, allowing for continuous alternating measurements of sample and reference CO_2_ gas on detectors configured to measure isotopologues of masses 44 to 49. Measurements taken with the Nu Perspective mass spectrometer (∼5 mg of sample material) consisted of three blocks of 20 cycles, totaling 1200 s of integration at a signal that ranged from 80 to 30 nA on mass 44 over the course of each acquisition. Carbonate standards ETH-1 and ETH-2 were used to perform a nonlinearity correction on sample unknowns. These nonlinearity corrected values were then projected into the I-CDES reference frame (*58*) using ETH-1-2, and ETH-3 along with four in-house standards. ETH-4 was not included in the corrections and was instead used as a check for data quality. Corrections were applied to tooth samples and standard data over a moving average of 10 standards on either side of a given sample and were calculated using Easotope software (*59*). Reconstructed temperatures were derived from the Δ_47_-temperature calibration of Anderson *et al*. (*60*)

$$\Delta_{47(\text{ICDES}\;90{}^{\circ}C}=0.0391\pm 0.0004\times 10^{6}/T{\left(K\right)}^{2}+0.154\pm 0.004$$

This equation was recently demonstrated to be statistically indistinguishable from a bioapatite-based calibration generated using the latest I-CDES standardized reference frame and therefore appropriate for application in bioapatite temperature reconstructions (*22*, *61*). The equation of Löffler *et al*. (*23*) was used to estimate the formation of waters during mineralization

$$1000\text{ln}\;\left(\alpha_{\text{apatite}-\text{water}}\right)=17.23\pm 0.59\times 10^{6}/T\left(K\right)-27.28\pm 1.73$$

Samples were prepared for phosphate δ^18^O analysis following the Rapid University of Chicago Dilute (i.e., Rapid UC) protocol (*62*, *63*). Approximately 1.0 mg of sample material was weighed out in 2-ml microcentrifuge tubes and dissolved overnight in 2.0 M nitric acid (HNO_3_). Calcium fluoride (CaF_2_) was then precipitated with the addition of 2.9 M hydrofluoric acid (HF) and 2.0 M sodium hydroxide (NaOH). Samples were centrifuged to pellet CaF_2_. The phosphate ion–containing supernatant was transferred to a new clean microcentrifuge tube. The CaF_2_ pellet was rinsed with 0.1 M sodium fluoride (NaF) to recover residual phosphate, and the resulting supernatant aliquot was combined with the first aliquot. Sample phosphate was precipitated as silver phosphate (Ag_3_PO_4_) with a silver amine solution containing 1.09 M ammonium hydroxide (NH_4_OH) and 0.37 M silver nitrate (AgNO_3_) at a pH range of 5.5 to 7.5. Adjustments to pH were made with the addition of small aliquots of 2 M HNO_3_ or 2 M NaOH, and samples were allowed to react for 10 min. Samples were centrifuged to pellet Ag_3_PO_4_ crystals and following the removal of supernatant were rinsed five times with distilled water and dried overnight in a 50°C oven. Dried samples were weighed out to ∼150 to 200 μg in packed silver capsules and analyzed in triplicate using a Thermal Conversion Element Analyzer (TC/EA)–ConFlow IV—Delta V plus a Continuous Flow Isotope Ratio Mass Spectrometer (Thermo Scientific, Bremen, Germany) in the Stable Isotope Ecosystem Laboratory of UC Merced (SIELO). Reference material NIST SRM 120C was precipitated and analyzed alongside sample unknowns as a reference material for quality control of the chemical procedure. The NIST SRM 120c yielded a δ^18^O value of 21.4 ± 0.3‰ (*n* = 6), comparing well with the average published value of 21.7 ± 0.5‰ (*64*). Reference materials USGS 80 (δ^18^O = 13.1 ± 0.2‰, *n* = 30) and USGS 81 (δ^18^O = 35.4 ± 0.3‰, *n* = 29) were used for linearity and drift corrections and in calibration to Vienna Standard Mean Ocean Water Scale.

#### FTIR analysis

We collected ATR-FTIR spectra for all fossil specimens using a Bruker Vertex 70 Far-Infrared Fourier Transform Infrared Spectrometer housed in the Nuclear Magnetic Resonance Facility at the University of California, Merced. We also collected spectra of modern alligator tooth enamel and Jurassic sauropod enamel that have previously been determined to be either well-preserved or diagenetically altered (*10*) as comparative samples. The spectra were collected from 400 to 4000 cm^−1^ over 32 scans at a spectral resolution of 4 cm^−1^. Each spectrum was background-corrected using a spline fit to several baseline points and slightly smoothed before index calculation. Data corrections and index calculations used custom R scripts modified from (*65*).

### Paleoclimate simulations

To explore the Maastrichtian climate and habitation for *T. rex* impact, we used a newly updated version of a state-of-the-art paleo-general circulation model and, for the first time, we apply a newly developed high-resolution version of the model, HadAM3B-N216-M2.1aD.

### Paleoclimate general circulation model

#### Low-resolution HadCM3L-M2.1D

Paleoclimate model simulations were carried out using a recent version of the UK MetOffice coupled Atmosphere-Ocean General Circulation Model (AOGCM), HadCM3 (specifically HadCM3L-M2.1D), following the nomenclature of Valdes *et al*. (*66*). HadCM3L-M2.1D has a model resolution of 3.75° longitude by 2.5° latitude in the atmosphere and ocean (∼250 km grid squares in the tropics), with 19 hybrid levels in the atmosphere and 20 vertical levels in the ocean with equations solved on the Arakawa B-grid. As is common in all climate models, sub-grid scale processes such as cloud, convection, and oceanic eddies are parameterized as they cannot be resolved at the scales required (usually meters to several kilometers) of the model resolution. Unlike contemporary modeling studies, global-scale soil texture, porosity, and albedo are unknown in the deep past. So a globally uniform medium loam soil characteristics in the model land surface scheme [MOSES 2.1; (*67*)] is used. The land surface scheme also includes evaporation from sub-grid scale lakes (which are prescribed as a lake fraction in each grid box, at the start of the simulation). We use a version of the model that includes the dynamical vegetation model TRIFFID (Top-Down Representation of Interactive Foliage and Flora Including Dynamics). TRIFFID predicts the distribution and properties of global vegetation based on plant functional types (PFTs), in the form of fractional coverage (and thus PFT co-existence) within a grid cell and which are in turn based on competition equations based on the climate tolerance of the five PFTs. However, due to a lack of geological spatiotemporal data recording, we use modern-day PFTs (broadleaf trees, deciduous trees, shrubs, C3 and C4 grasses). This is a common practice in paleoclimate modeling and is not expected to adversely affect the Maastrichtian simulation.

The model has a further update that includes modification to cloud condensation nuclei (CCN) density and cloud droplet effective radius following the work of Sagoo *et al*. (*68*) and Kiehl and Shields (*69*). This raises higher latitude temperatures without significantly changing tropical temperatures, thereby reducing pole-to-equator temperature gradient with higher latitude temperatures better in line with proxy observations. This update is also found to work under both hot, cool, and icehouse conditions, as well under pre-industrial boundary conditions, making it appropriate for use across the Phanerozoic (*36*, *70*).

The ocean model is based on the model of Cox and Bryan (*71*) and is a full primitive equation, three-dimensional model of the ocean. A second order numerical scheme is used along with centered advection to remove nonlinear instabilities. Flux adjustments [such as artificial heat and salinity adjustments in the ocean component model (*72*) to prevent it from drifting to unrealistic values] are not required in this model, which is crucial for long paleoclimate simulations (*73*). Sea ice is calculated on a zero-layer model with partial sea-ice coverage possible with a consistent salinity assumed for ice.

Each simulation was initialized from an equilibrated pre-industrial state in the atmosphere and ocean. Surface vegetation was uniformly set as shrub everywhere and allowed to evolve through the interactive dynamic vegetation sub-model, TRIFFID (called every 10 model days), in response to the evolving local climate.

The HadCM3 family of models has extensively contributed to the Coupled Model Intercomparison Project (CMIP 1–5) experiments, as well as the Paleoclimate Modeling Intercomparison Project (PMIP 1–4), demonstrating skill at reproducing the modern-day climate (*66*) and an array of paleoclimate experiments (*74*, *75*). Paleoclimate experiments require in the order of hundreds of years to reach a near-surface equilibrium state but substantially longer (many thousands of years) for the deep ocean (*73*), and true climate equilibrium, due to the long period of adjustment of ocean circulation to applied forcings. Lower-resolution models are less computationally expensive, allowing fully equilibrated simulations of deep-time climate to be undertaken, which would not be possible with higher-resolution, more complex coupled CMIP6 generation models. For the low-resolution coupled simulation run in this study, over 20,000 model years were required to reach a fully equilibrated state in the deep ocean (and by extension global circulation).

#### High-resolution HadAM3B-N216

Two simulations with the high-resolution atmosphere-only HadAM3B-N216-M2.1aE paleoclimate model comprising a resolution of 0.83° by 0.56° longitude by latitude (∼60 km grid box) with 30 levels in the atmosphere for the Maastrichtian were conducted. HadAM3B-N216-M2.1aE is exactly the same as the atmospheric component model of HadCM3BL-M2.1aD (300-km grid box), but at higher spatial resolution. No changes to the core physics are made and kept the same as the lower-resolution version of the model to ensure that horizontal and vertical resolution changes are the only significant difference. Some resolution-dependent parameters were modified, such as “RCrit,” part of the convection scheme that determines when convection is triggered. This value was increased in all 30 atmospheric levels, except the two lowest, in line with other climate models of a similar resolution, while the threshold value for cloud liquid water formation was also increased. MPARWTR (moisture parameter), which controls the phase change of water partitioning during convection, was decreased by a factor of one. Owing to the increase in topographic detail, the gravity wave drag scheme was also modified due to the increased vertical gradients and roughness of the higher-resolution digital elevation model (DEM).

Climatological (last 100 years of the run) SSTs and sea-ice fields were calculated from the low-resolution version of the Maastrichtian simulations at 3× CO_2_ and 6× CO_2_, respectively, and used as a boundary condition in the model. The interactive vegetation scheme TRIFFID is run in equilibrium mode and “accelerated.” TRIFFID runs for 50 years of TRIFFID every one model year of the run, where fluxes between the land and the atmosphere are calculated during that year. This allows for a quick spin-up of the vegetation and soil carbon that would otherwise take 500 to 1000 model years.

### Maastrichtian snapshot simulation boundary conditions

Paleogeographic DEMs (topography, bathymetry, and land-sea distribution) were produced by Robertson Plc (fig. S5). Each geologic stage and time-specific DEM are interpolated from a 0.1° by 0.1° grid onto the HadCM3L 3.75° by 2.5° grid and 0.83° by 0.56° longitude by latitude grid, respectively. There is no land ice in this time period. *P*co_2_ (partial pressure of CO_2_) is set at 840 and 1680 ppmv for both high- and low-resolution simulation, both constraining the upper and lower uncertainty bounds from proxy reconstructions (*76*) at 70 Ma. Time-specific solar luminosity for each simulation was based on Gough (*77*). All other boundary conditions (e.g., orbital parameters) are held constant at pre-industrial values. To ensure that each simulation is fully adjusted to the boundary conditions, we follow a three-stage spin-up protocol so that each simulation is fully equilibrated. (i) The globally and volume-integrated annual mean ocean temperature trend is less than 1°C per 1000 years, (ii) trends in surface air temperature are less than 0.3°C per 1000 years, and (iii) net energy balance at the top of the atmosphere, averaged over a 100-year period at the end of the simulation, is less than 0.25/W m^2^. In general, these simulations have been run for over 9000 model years to ensure full Earth system equilibrium. Climate means were produced from the last 100 years of each simulation. SSTs and sea ice from each HadCM3BL-M2.1aD equilibrated run were used as boundary conditions for the high-resolution, HadAM3B-N216-M2.1aE, set of simulations.

Climate means (i.e., mean annual temperature, mean annual precipitation, warm month mean, and cold month mean) were calculated from the last 100 years of each simulation to avoid the influence of interannual variability. For the high-resolution model, the last 30 years of each run were used to calculate climatological means.

### Wet-bulb temperature calculation

A more potent measure of heat stress than surface air temperature alone can be measured through the combined interplay of temperature and moisture, known as wet-bulb temperature. Wet-bulb temperature is a measure of how hot it feels when taking into account both the ambient surface air temperature and humidity. It is an important heat stress metric because it describes the physiological limit for a species and, once surpassed, will lead to death. For endotherms, a flux of internal body heat to the surrounding environment is required to stop the body from overheating and damaging internal organs through sweating or panting (evaporative cooling). Today, for most endotherms, if ambient wet-bulb temperatures rise above 35°C for sustained periods (hours-days), survivability will be compromised (*78*). In our Maastrichtian simulations, this threshold is never passed (fig. S7), suggesting that *T. rex* habitability was not constrained by moist heat stress. Wet-bulb temperature (*T*_w_) is derived from a more accurate computation from Davies-Jones (*79*) (their equation 3.8) with equivalent potential temperature calculated from Bolton (*80*) (their equation 38). Wet-bulb approximations such as that of Stull (*81*) are not desirable for us as it is calibrated for the modern day and does not apply to warmer time periods. The complete derivation can be found in appendix A of (*82*). *T*_w_ values of ≥35°C are assessed to be critically lethal and lead to mortality (*83*).

### Habitat suitability modeling using a virtual species approach and inferred thermophysiology for *T. rex*

To quantitatively assess the Maastrichtian climatic niche and potential habitation for a hypothetical apex predator analogous to *T. rex*, we used a virtual species modeling approach using the VirtualSpecies package in R (*84*). Environmental suitability was generated by integrating multiple climatic variables derived from high-resolution paleoclimate simulations (see paleoclimate methods). We used seasonal paleoclimate output from the general circulation model, specifically cold month mean temperature (cmm), warm month mean temperature (wmm), and indices of dry and wet months (drymon, wetmon). These variables were extracted from high-resolution Maastrichtian simulations, rotated from 0° to 360° to −180° to 180° longitude, assigned a WGS84 geographic coordinate system (EPSG:4326), and cropped to a North American analysis window (−167° to −20° longitude, 10° to 90° latitude). Paleogeographic context was provided by a 67-Ma GPlates (*85*) reconstruction of the Robertson model, from which we generated a polygonal representation of the Maastrichtian North American landmass; these coastlines were rasterized and used to spatially mask the paleoclimate fields. To more realistically represent uncertainty associated with highly contentious late Maastrichtian paleocoastlines of the Western Interior Seaway (WIS) (*86*), we additionally incorporated the geography (land-sea) mask from the climate model (qrparm.mask), resampled it onto the climate grid, and treated cells classified as “sea” (mask = 0) as climatically unsuitable. For each climate experiment, we computed two separate environmental stacks: a “GPlates-only” version masked solely by the reconstructed GPlates continent polygons, and a “geography-mask” version additionally constrained by the model land-sea mask to incorporate coastline variability imposed by the WIS; continuous suitability surfaces were generated for both and combined into a “highstand” coastline scenario by averaging suitability values on a per-pixel basis, assigning a value of zero to cells that were covered by epicontinental sea in the geography mask. At that time (∼68 to 66 Ma), the sea level of the WIS was undergoing forced regression (*87*, *88*), resulting in a coastline configuration that closely resembles the one shown in Fig. 3. We therefore examined both a regressive (lowstand) configuration and a higher WIS (highstand) scenario using alternative climate simulations, and project suitability patterns for each as well as for the averaged “middle-ground” coastline.

#### Modeling the thermal niche of T. rex

We developed a novel thermal suitability function that converts environmental temperature (°C) for seasonal variables (cmm, cold month temperature and wmm, warm month temperature) into a normalized suitability index ranging from 0 (unsuitable) to 1 (optimal). This transformation was built around empirically defined thermal limits of −13°C (minimum) and 43.6°C (maximum) for endotherms (*52*). The GlobTherm dataset, including thermal preferences for >2000 living metazoan animals, was vetted to include only true avian and mammalian endotherms (e.g., removing monotremes and some marsupials and bats, which are known heterotherms). A subset dataset of 465 homeothermic endotherms was obtained (data S3) and analyzed for basic statistics (mean, max, min) of their thermal ranges. From the isotopic investigation in this study, a thermal optimum (or “relaxed” plateau) occurring within the range of 34.7° to 37.3°C and peaking at 36.3°C was inferred for *T. rex* and used to anchor the central portion of the suitability curve. Using these metrics, a function was constructed as a piecewise quadratic curve with the following components (Fig. 3A, see main text): left segment (*x* < 34.7°C): a shallow quadratic function gradually increases suitability from the extreme low of −13°C to the start of the relaxed central region (34.7°C), ensuring continuity with the central segment; a central (relaxed) segment (34.7° to 37.3°C): in this range, the function is defined by a gently curved quadratic that peaks at 36.3°C with a maximum value of 43.6°C. The low curvature in this segment creates a “relaxed” optimum, reflecting a broad thermal neutral zone within which physiological performance remains high; a right tail segment with a decline in suitability above the relaxed region was modeled in two parts. A first quadratic segment spans from 37.3°C to 45°C; here, the curve is made slightly steeper than the central segment to begin reducing suitability under increasing thermal stress. A second, even steeper quadratic segment was then applied from 45°C to 48°C, where suitability rapidly falls, with temperatures above 48°C being considered entirely unsuitable (mapped to −13°C in the original scale). The continuous output of this piecewise function originally spans from −13 to 43.6, conforming to the entire thermal range of modern endotherms. This range was linearly normalized to yield a suitability score ranging from 0 to 1.

#### Modeling the precipitation component

Following the procedure of modeling the abiotic niche of other extinct dinosaurian taxa (*5*, *6*, *89*), seasonal precipitation patterns were incorporated by modeling the “drymon” and “wetmon” layers with logistic functions (parameterized with α = 2 and β = 1), such that areas receiving precipitation above 1 mm day^−1^ receive higher suitability scores. This represents a loose constraint on precipitation dependence and aligns with previous niche modeling results on dinosaurian taxa (*5*, *6*, *89*). This abiotic niche-habitat suitability curve was implemented as a logistic response of the form 1/(1 ± exp[−*k*(rain − threshold)]), with *k* = 2 and threshold = 1 mm day^−1^, and was assigned to both drymon and wetmon layers in VirtualSpecies via the same formatFunctions interface used for the thermal responses.

#### Data processing and virtual species implementation

Environmental raster datasets were processed using the terra package (*90*) to mask and crop the high-resolution paleoclimate simulations to the region of interest. GPlates-derived Maastrichtian coastlines (reconstructed at 67 Ma using the Robertson model) were converted from vector to raster form and used to define a North American analysis mask, which was intersected with the model land-sea mask; this ensured that suitability was only evaluated on emergent land in each paleogeographic scenario. All climatic variables (cmm, wmm, drymon, wetmon) were stored as multi-layer SpatRaster objects, rotated to a −180° to 180° longitude convention, and given a global extent of −180° to 180° and −90° to 90° before cropping to the North American window. For each coastline treatment (GPlates-only and geography-mask), we constructed an environmental stack and passed it to VirtualSpecies::generateSpFromFun, specifying our custom thermal and precipitation response functions via formatFunctions, with species.type set to “additive” and rescale.each.response = TRUE so that each environmental response was first rescaled to [0, 1] and then combined additively to yield an overall suitability surface. The two resulting continuous suitability rasters (one for the GPlates-only coastline and one constrained by the geography mask) were then combined into a “highstand” surface by averaging per-pixel suitability, after recoding cells that were ocean in the geography mask to zero; this approach down-weights areas that are only intermittently emergent across paleogeographic reconstructions, particularly along the margins of the WIS.

For some analyses, we additionally converted continuous suitability to presence-absence using VirtualSpecies::convertToPA (*84*) with a probabilistic threshold parameter (β = 0.65), providing a binary realization of potential occupancy that complements the continuous suitability maps. The resulting continuous, climatically constrained suitability maps were then used as the basis for overlaying fossil occurrence data to infer potential late Maastrichtian distribution for *T. rex*. *Tyrannosaurus* occurrences (https://paleobiodb.org/data1.2/occs/list.csv?base_name=Tyrannosaurus&interval=Maastrichtian&cc=NOA&pgm=gplates,scotese,seton&show=full,classext,genus,subgenus,acconly,ident,img,etbasis,strat,lith,env,timebins,timecompare,resgroup,ref,ent,entname,crmod) were screened and vetted for accuracy (*91*), plotted in paleocoordinates within the same reference frame as the climate simulations via palaeoverse R package (*92*), and high-suitability regions (e.g., ≥0.5) were visualized using contour lines superimposed on rasters of the suitability surface to emphasize the core of the mechanistically modeled niche. This integrated framework allowed us to flexibly represent the thermal niche of the hypothetical apex predator (*T. rex*) while explicitly considering the physiological impacts of both temperature and moisture regimes during the Maastrichtian, and to avoid propagating paleogeographic uncertainty by comparing suitability patterns across alternative coastline and WIS configurations.

## Supplementary Materials

### The PDF file includes:

Supplementary Text Figs. S1 to S7 Legends for data S1 to S3 References
