# Supplementary material for: The body temperature of Tyrannosaurus rex
Source: Sci Adv. 2026 Sep 16;12(38):eaeb7653. doi: 10.1126/sciadv.aeb7653 (PMC13580596; doi:10.1126/sciadv.aeb7653)
Supplement: Supplementary file 1 [file sciadv.aeb7653_sm.pdf]

Science Advances
AAAS

Supplementary Materials for

The body temperature of *Tyrannosaurus rex*

Randon J. Flores *et al.*

Corresponding author: Randon J. Flores, rflores9@ucla.edu; Robert A. Eagle, robeagle@g.ucla.edu

Sci. Adv. **12**, eaeb7653 (2026)
DOI: 10.1126/sciadv.aeb7653

The PDF file includes:

Supplementary Text
Figs. S1 to S7
Legends for data S1 to S3
References

Other Supplementary Material for this manuscript includes the following:

Data S1 to S3

Supplementary text

Description of fossils and collection sites

Hell Creek Formation

All specimens were collected in the Late Cretaceous Hell Creek Formation of the United States. The Hell Creek formation is an upper Maastrichtian sedimentary sequence composed predominantly of fluvialites deposited on a coastal plain bordering the Western Interior seaway (*92*). The formation is notable for sourcing numerous well-recognized dinosaur fossils, as well as the sedimentary layer marking the end of the Cretaceous at the K-Pg boundary. Outcrops of the Hell Creek Formation can be found in Montana, Wyoming, and North and South Dakota.

Tyrannosaurus rex

Two teeth identified as belonging to *Tyrannosaurus rex* specimen LACM 150167 (nicknamed "Thomas") were provided by the Los Angeles Museum of Natural History. Additionally, one isolated tooth also identified as *Tyrannosaurus rex* but not belonging to Thomas was provided (LACM 151468). The two teeth belonging to specimen LACM 150167 (LACM 150167 EEEE3, LACM 150167 PPPP) and the isolated partial tooth LACM 151468 were collected from deposits of the Hell Creek Formation near Ekalaka (Montana, USA) by expeditions from the Natural History Museum of Los Angeles County, directed by Luis M. Chiappe, between 2003-2005. Teeth from LACM 150167 were found in close association with a *T. rex* skeleton (LACM 150167); LACM 151468 is morphologically indistinguishable from these or other T. rex teeth. The latest Cretaceous age of the Hell Creek Formation is extremely well-established—the specimens come from near the stratigraphic boundary between this formation and the overlying Paleocene Fort Union Formation. All these specimens are housed in the Dinosaur Institute of the Natural History Museum of Los Angeles County (https://nhm.org/research-collections/departments/dinosaur-institute), whose collections are fully accessible to qualified researchers worldwide. The skeleton was disarticulated but deemed to be well preserved. Body size estimates based on femur size place Thomas at approximately 15% smaller than the largest T. rex specimens discovered, such as Sue (FMNH PR 2081) while bone histology yielded an approximate young adult age range.

Crocodilians

Five teeth assigned to a non-specific crocodilian were provided by the Raymond M. Alf Museum of Paleontology (three teeth from RAM 21150, and two teeth from RAM 21157). The teeth sampled possess incrassate morphology and apicobasal ridges typically associated with the genus *Borealosuchus,* but were not identified at the time of curation at a taxonomic level more specific than belonging to a crocodilian. Crocodilian teeth were collected from the vertebrate fossil site UCMP V85092 in McCone County, Montana. The locality is a local lag deposit composed of sandstone with ironstone concretions. It should be noted that specimens collected from this site are likely not precisely contemporaneous with Thomas, given the distance and difference in stratigraphic depth between the fossil localities. Vertebrate fossil site UCMP V85092 is part of a

Lancian age deposit in the K-Mark Channel fill which is approximately 20 meters below the K-Pg boundary (*93*). We therefore approximate the environmental conditions experienced by the respective taxa as "latest Cretaceous" when making observations based on environmental temperature recorded by crocodilian teeth.

<u>Assessment of diagenesis</u>

We assessed diagenesis using four methods (discussed below). Briefly, *T. rex* and crocodilian enamel show distinct carbon isotope values consistent with preservation of ecological signals, distinct enamel and dentin bulk isotope compositions, PO_4–CO_3 $\delta^{18}O$ offsets that are not clearly indicative of diagenetic alteration, and spectroscopic methods do not indicate substantial recrystallization of enamel mineral or large amounts of structural carbonate during fossilization.

Dietary $\delta^{13}C$ relationships

Fossils collected from the Hell Creek Formation have also been shown to exhibit good preservation through the recording of presumed dietary signals in isotopic signatures, which show niche partitioning among dinosaurs with differing ecological roles (*26*). The offset in carbon isotopes observed from T. rex and crocodilian specimens from this study and those published in Fricke et al. (*26*) show a pattern similar to that observed in the Oldman Formation, another late Cretaceous fossil assemblage (*27*), wherein Tyrannosaurid ^{13}C is depleted relative to herbivorous ornithischian dinosaurs indicating an apparent predator-prey relationship (Fig. S1). Similarly, crocodilians measured in this study show lower $\delta^{13}C$ relative to gar reported in Fricke et al. (*26*), while a single measurement taken from a champsosaurus tooth (RAM 21143) for this study is further depleted in ^{13}C once again mirroring expected relationships (*27*). The preservation of dietary signals in carbonate $\delta^{13}C$ suggests that original isotopic composition is preserved in these specimens and not uniformly reset as might be expected in the case of large-scale diagenetic overprinting.

Comparison of enamel and dentin isotopic composition

The enamel and dentin components of vertebrate teeth are expected to exhibit distinct isotopic signatures as a result of the elevated susceptibility to diagenetic alteration in dentin relative to enamel (*25*). Fig. S2 shows that dentin sampled from LACM 150167 EEEE 3 as well as LACM 151468 show a distinct signature relative to enamel measurements, falling outside the 95% confidence ellipse calculated for all other enamel samples.

PO_4–CO_3 $\delta^{18}O$ offset

The offset between measured $\delta^{18}O$ of tooth enamel carbonate and phosphate is commonly used as a tool for assessment of diagenesis (*25*). Recent work has called into question the reliability of PO_4–CO_3 $\delta^{18}O$ offsets as an indicator of diagenetic alteration due to uncertainty regarding the influence of tooth mineralization processes (*32*, *33*) as well as diet and physiology (*34*, *94*). Furthermore, published offsets cited in assessments of diagenesis of terrestrial vertebrate fossils are based almost exclusively on mammalian apatites meaning additional work will be required to

confirm whether reptilian teeth actually follow the same trend as that observed in mammals. However, this method still provides useful information for identifying signs of chemical alteration through comparison with the full range of existing vertebrate data (Fig. S3). Modern mammal apatite shows an overall range of 6.6 to 10.6 ‰ (*32*). Measurements from both teeth from LACM 150167 and 151468 fall within this range at 10.4 ± 0.5 ‰ (1 SD) for LACM 150167 EEEE 3, 10.4 ± 0.5 ‰ (1 SD) for LACM 150167 PPPP, and 9.8 ± 1.2 ‰ (1 SD) for LACM 151468. In contrast, measurements of phosphate and carbonate $\delta^{18}O$ in *T. rex* bones from the Hell Creek Formation can vary by up to 17 ‰ (*29*), well outside the range observed in any modern vertebrate (*25*). Our results are therefore consistent with observations that tooth enamel preserves an isotopic composition closer to that expected for vertebrate skeletal tissues than dentin or bone. Offsets for crocodilians from this study are more variable, ranging from 8.4–11.7 ‰. While the 11.7 ‰ value obtained from 2 crocodilian teeth is higher than the ~9 ‰ value typically considered indicative of well-preserved samples, this value is based on mammalian skeletal apatites and therefore precipitation at mammalian body temperatures. The variability in crocodilian tooth PO_4–CO_3 $\delta^{18}O$ spacing can potentially be explained by the difference in body temperatures and physiology between crocodilians and mammals (*49*, *94*) as well as variations in climate and water sources (*34*). The phosphate component of bioapatite is more sensitive to seasonal changes in $\delta^{18}O$ leading to differences in the PO_4–CO_3 $\delta^{18}O$ spacing at different periods of tooth formation (*32*), and the HCF is known to exhibit a wide range of environmental water $\delta^{18}O$ values due to variable water source inputs (~ -6–20 ‰ (*37*, *95*)). For this reason, we interpret the other diagenetic screening parameters listed here as stronger indicators of original isotopic preservation in fossil samples.

FTIR analysis

Fourier Transform Infrared spectroscopy (FTIR) has been used to assess preservation in fossil samples of bone and tooth apatite (*35*, *96–98*). FTIR measurements are used to investigate diagenetic recrystallization and preferential loss of carbonate in fossil specimens by characterizing chemical functional groups (i.e., PO_4, CO_3) within the bioapatite mineral lattice which may become altered from their original state through the process of fossilization and comparing them to data from in modern analogues (*35*, *71*); (Fig. S4). These functional groups are measured as absorption spectra with varying peak intensities corresponding to the abundance of that group in the sample. These peaks can be compared between samples by calculating unitless indices, which represent the ratio of spectral peaks to troughs at defined absorption bands. The B-carbonate-on-phosphate index (BPI) index describes the B-site carbonate, or carbonate occupying the phosphate location of the lattice, which makes up the majority of carbonate present in the apatite lattice and is therefore representative of the overall carbonate content (*71*). The A-carbonate-on-phosphate index represents the A-site carbonate, or carbonate in the Hydroxyl location (*99*). Also of note is the Phosphate-crystallinity index (PCI), which is related to the overall crystallinity and crystal size of the mineral (*99*). Although direct comparisons between FTIR measurements performed on different instruments with different

analytical (ATR vs Transmission mode) and data reduction methods, general trends can be resolved in comparison of fossil materials (*96*). Previous studies have shown that API decreases with sample age while PCI typically increases with age (*98*, *99*). The decrease of A-site carbonate has been proposed to occur as a result of increased solubility where the carbonate has been substituted into the apatite mineral lattice, or by replacement of A-site carbonate with fluorine (*99*). The loss of A-site carbonate may result in altered isotopic values if A-site carbonates are isotopically distinct from B-site carbonates.

Overall, FTIR indices from our *T. rex* specimens are intermediate between modern alligator enamel and Jurassic sauropods enamel and are dissimilar to the altered Jurassic enamel. (Fig. 4). The altered Jurassic Camarasaurus enamel of the specimen (DMN CAM33) with the highest PCI, and the lowest BPI and API values, has previously been determined to be heavily diagenetically based on anomalously high temperatures and anomalous carbon and oxygen isotope compositions. The Hell Creek specimens have PCI and API values intermediate between the modern alligator enamel value and the older Jurassic teeth. Both the Hell Creek and the single well-preserved Jurassic tooth enamel sample yielded BPI values higher than the altered sauropod teeth and closer to the modern alligator. When comparing FTIR indices to offsets between PO_4 and CO_3 $\delta^{18}O$, the Jurassic samples yield lower PO_4-CO_3 $\delta^{18}O$ offset values than those from the Cretaceous Hell Creek Formation. The sample with the highest offset between PO_4 and CO_3 $\delta^{18}O$ does not exhibit a clearly distinct index value from any of the other Hell Creek Formation samples, and while it yielded the lowest BPI value of those samples, it is nearly identical to the well-preserved Jurassic sauropod sample. Lastly, all teeth for which FTIR measurements were performed show carbonate contents well within the expected range of modern vertebrates (*100*) and in close agreement with modern reptile teeth specifically (*35*) (Fig. S3), which further suggests preservation of original material.

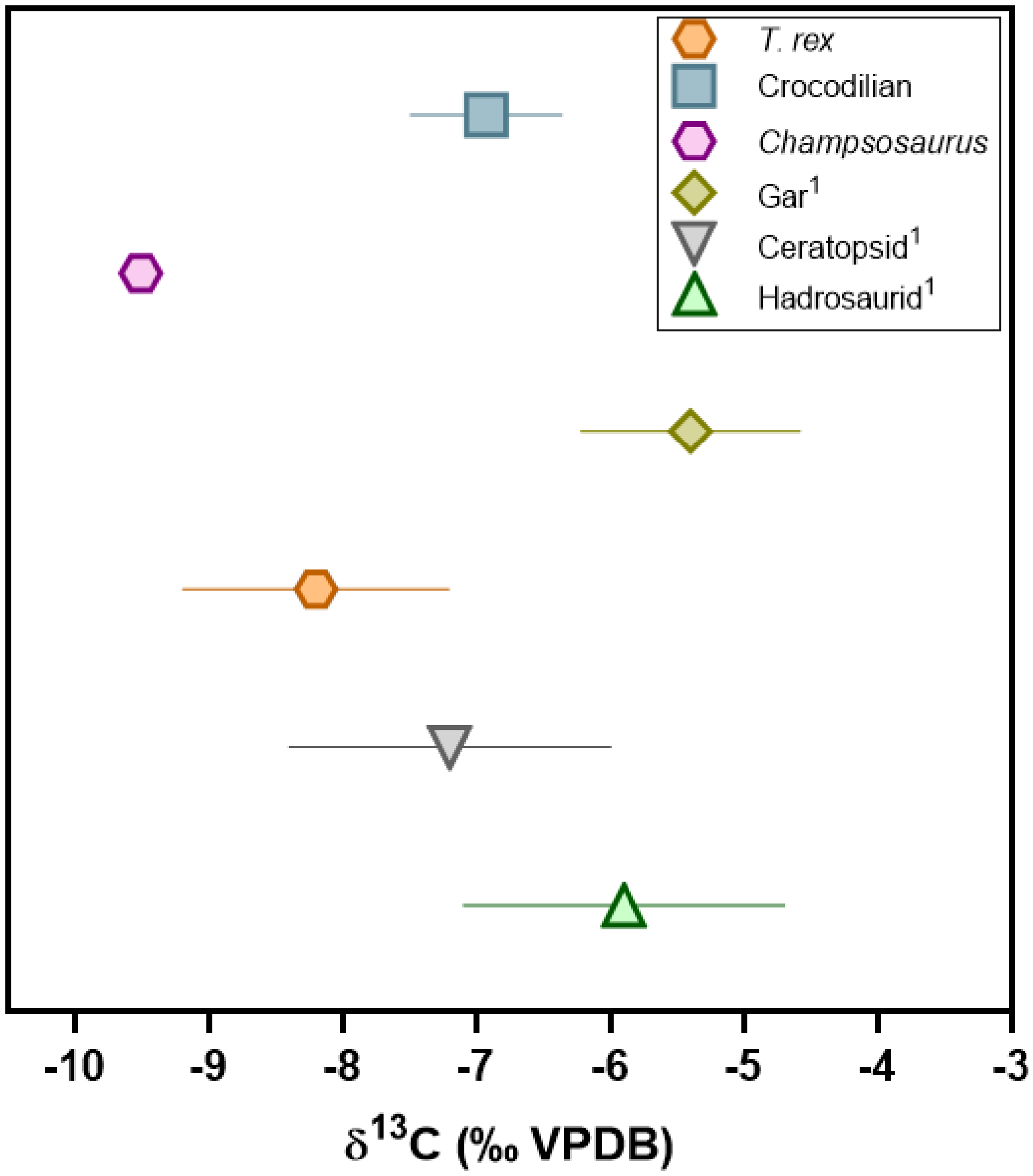

Fig. S1. Comparison of fossil tooth carbonate $\delta^{13}C$ showing preservation of dietary offsets between predator and prey taxa.

A single replicate from the tooth of *Champsosaurus* is included here for further comparison of aquatic taxa but due to low replication this sample is only used in the context of this diagenetic screening approach and not included in temperature comparisons. [1](*26*)**.**

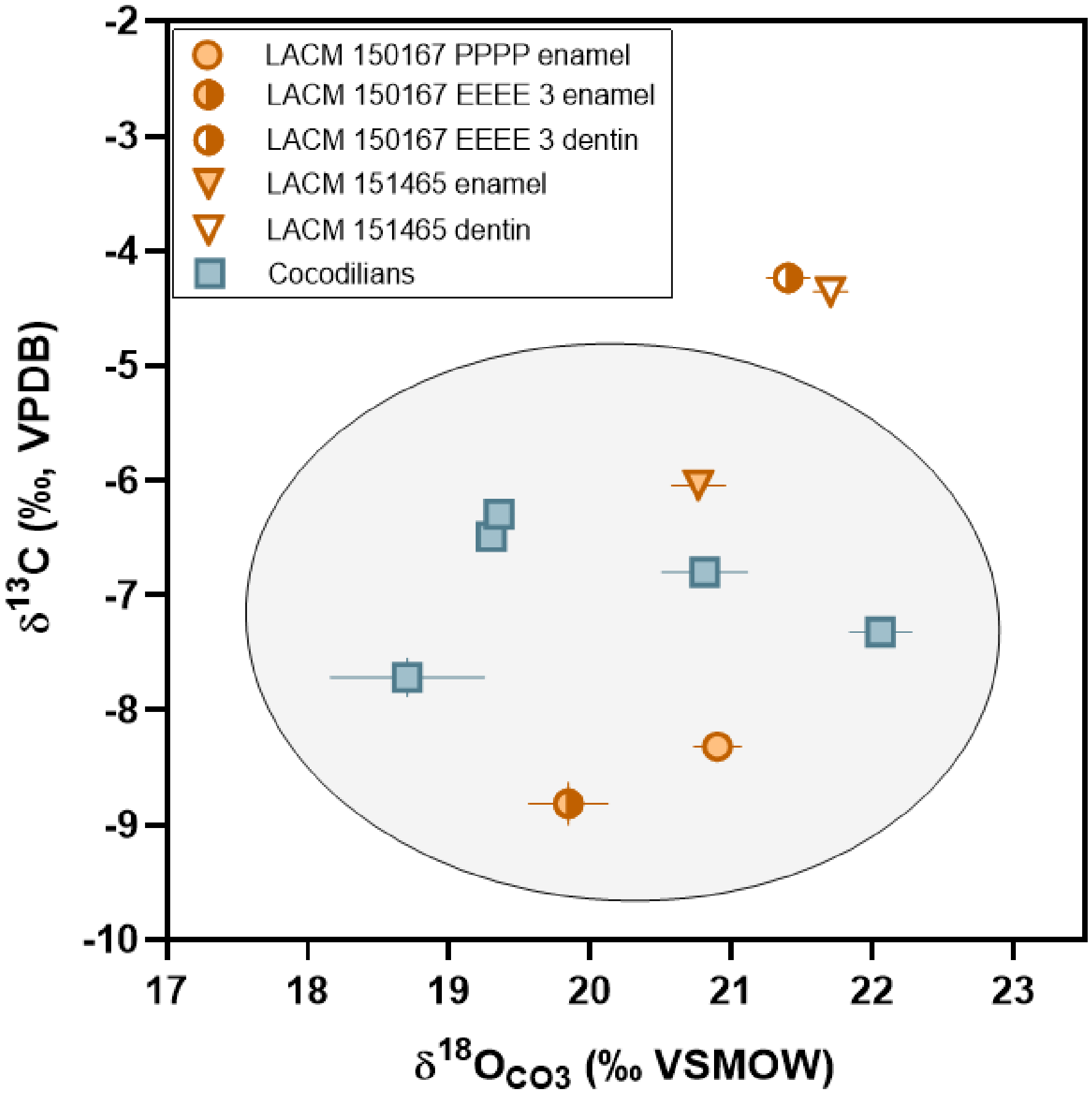

Fig. S2. Comparison of tooth carbonate $\delta^{18}O$ and $\delta^{13}C$. Gray shaded circle indicates 95% confidence ellipse of enamel samples.

While no dentin measurements were made for crocodilian specimens, all enamel samples plot within a range distinct from the dentin specimens analyzed which fall outside this 95% confidence ellipse. Only one tooth from LACM 150167 was measured to preserve precious material from fossil specimens assuming that diagenetic effects would affect both teeth in a similar manner.

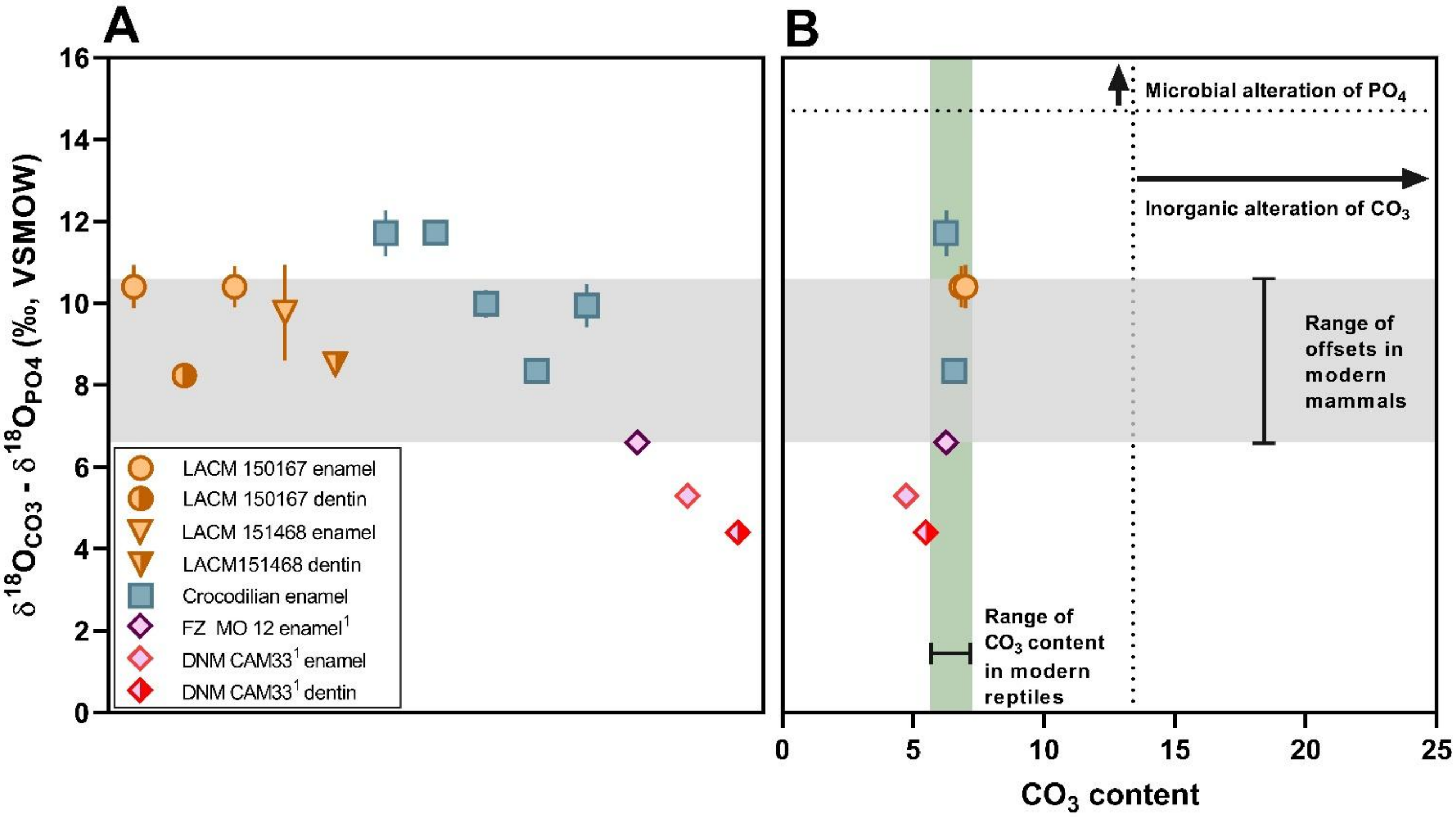

Fig. S3. Comparison of phosphate and carbonate δ^{18}O spacing in tooth enamel and dentin samples.

A) Samples FZ CA MO12 and DNM CAM33 are *Camarasaurus sp*. specimens previously reported in Eagle et al. (*10*), where DNM CAM33 was determined to be geochemically altered. B) Comparison of phosphate and carbonate δ^{18}O spacing with carbonate content determined based on FTIR measurements of B site carbonate. Vertical dotted line indicates the range of carbonate content observed in vertebrate bioapatite, horizontal dotted line indicates the upper limit of offsets between phosphate and carbonate δ^{18}O observed in modern vertebrates (*74*). FTIR measurements were not performed on LACM 151468 or HCF croc 2, 3, and 5 (see data S2).

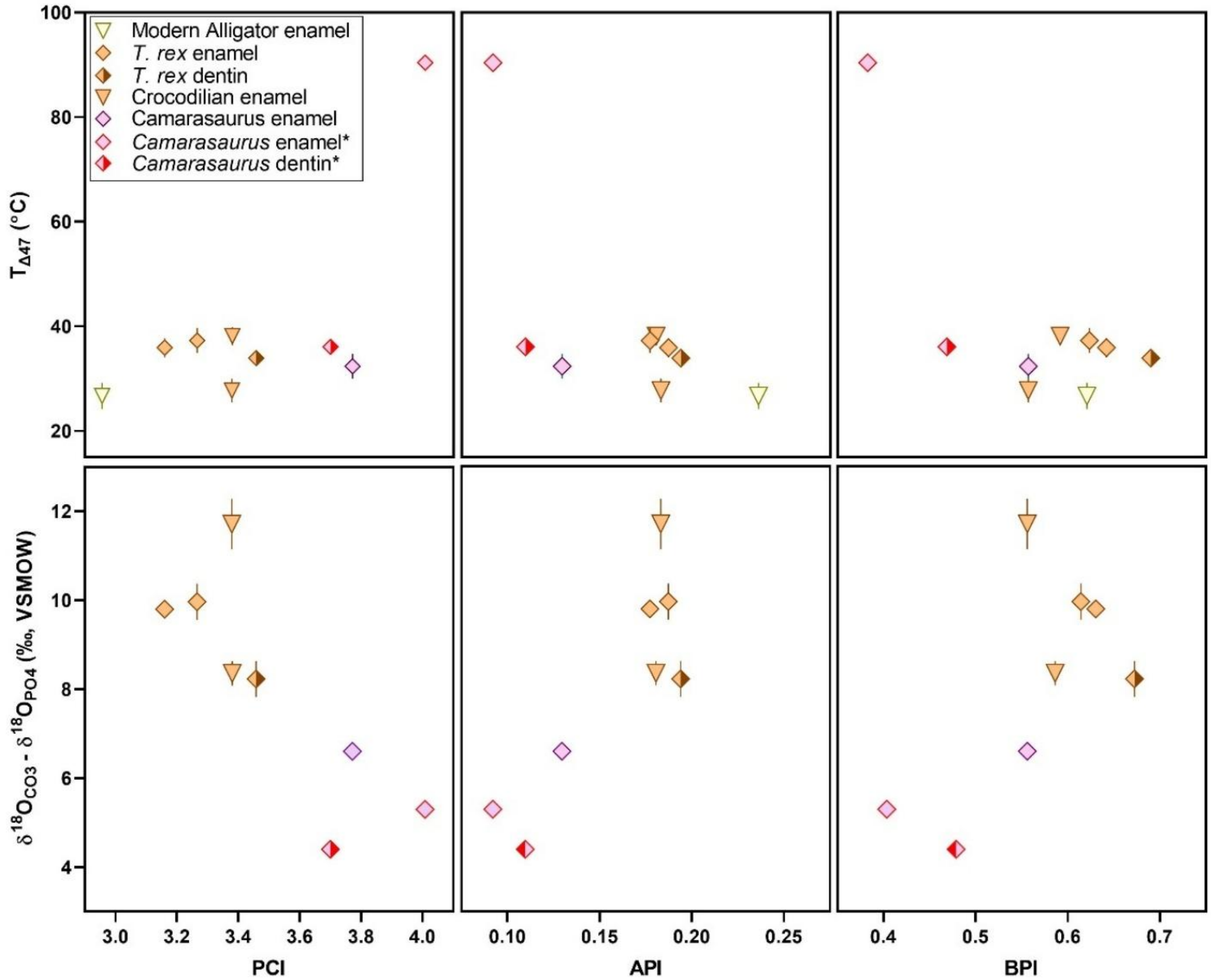

Fig. S4 Comparison of FTIR indices calculated for fossil teeth

Data show results for teeth from this study as well as Jurassic sauropod teeth reported in Eagle et al. (*10*) and a modern alligator tooth from Eagle et al. (*28*). No PO_4 measurements for the modern alligator were available from Eagle et al. (*28*) and are therefore not included in figures comparing FTIR indices to PO_4–CO_3 $\delta^{18}O$ spacing. Samples outlined in red were deemed to be chemically altered and excluded from their respective publication. (PCI - Phosphate-Crystallinity Index; BPI - B-carbonate-on-Phosphate Index; API - A-carbonate-on-Phosphate Index)

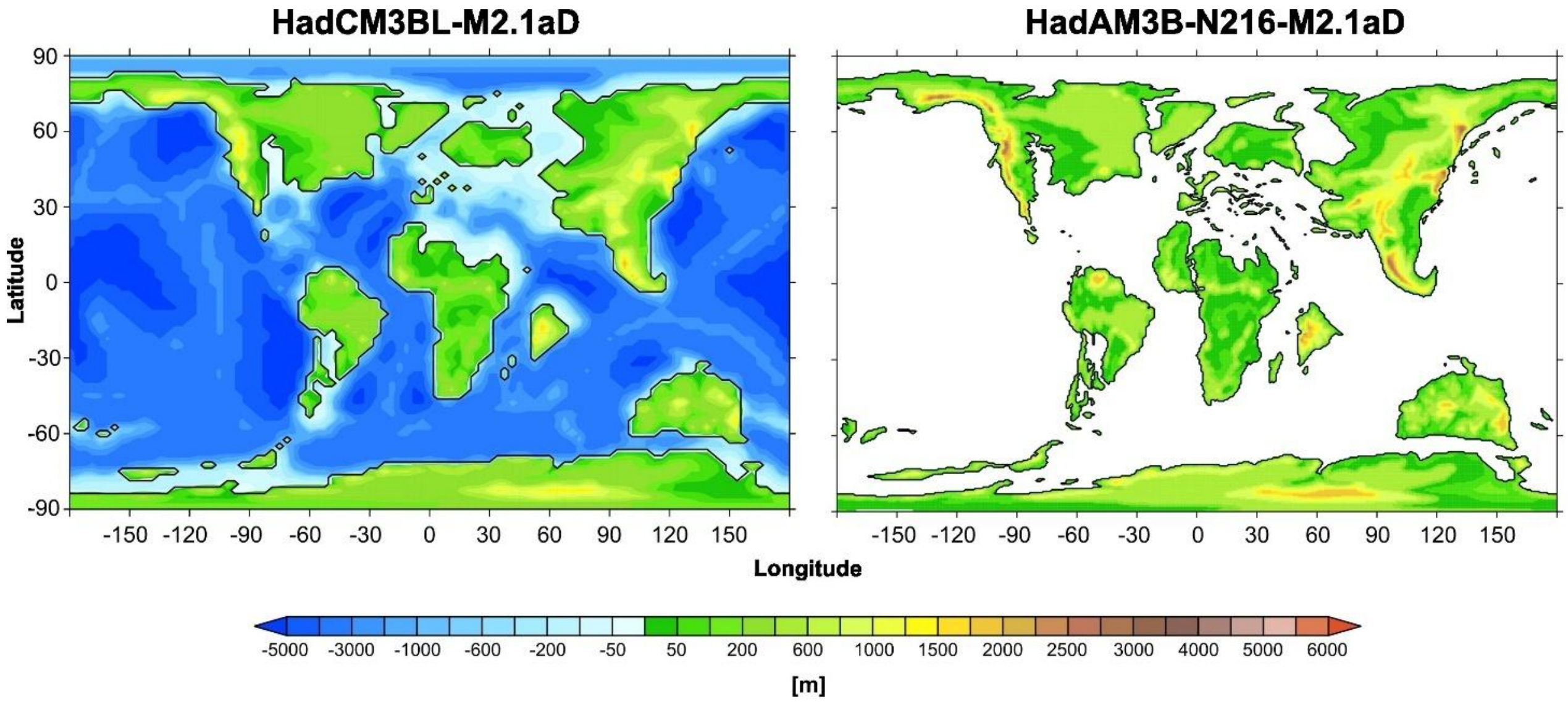

Fig. S5. **Low and high resolution paleogeography (m) used in climate models.**
The Robertsons Plc. paleogeography (bathymetry, topography, land-sea distribution) for the coupled atmosphere-ocean low-resolution model (left). The high-resolution paleogeography (topography, land-sea distribution) for the atmosphere-only high-resolution model (right).

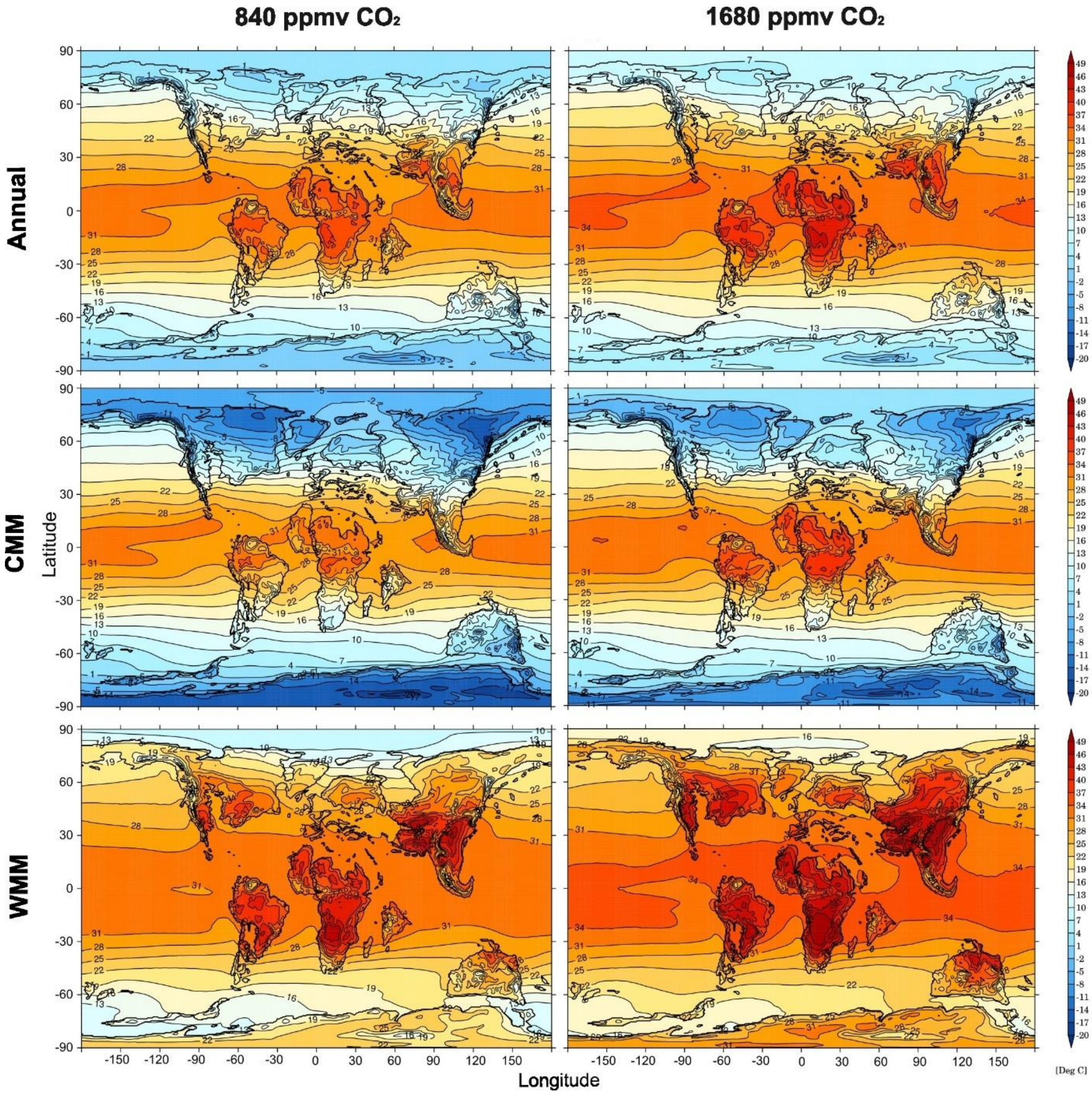

Fig. S6. **Simulated air temperatures for the Maastrichtian.**
High-resolution model simulated temperatures for the Maastrichtian at $3xCO_2$ (left column) and $6xCO_2$ (right column) showing 1.5m mean annual air temperatures (°C), 1.5m Cold Month Mean (CMM) air temperature (°C), 1.5m Warm Month Mean (WMM) air temperature (°C).

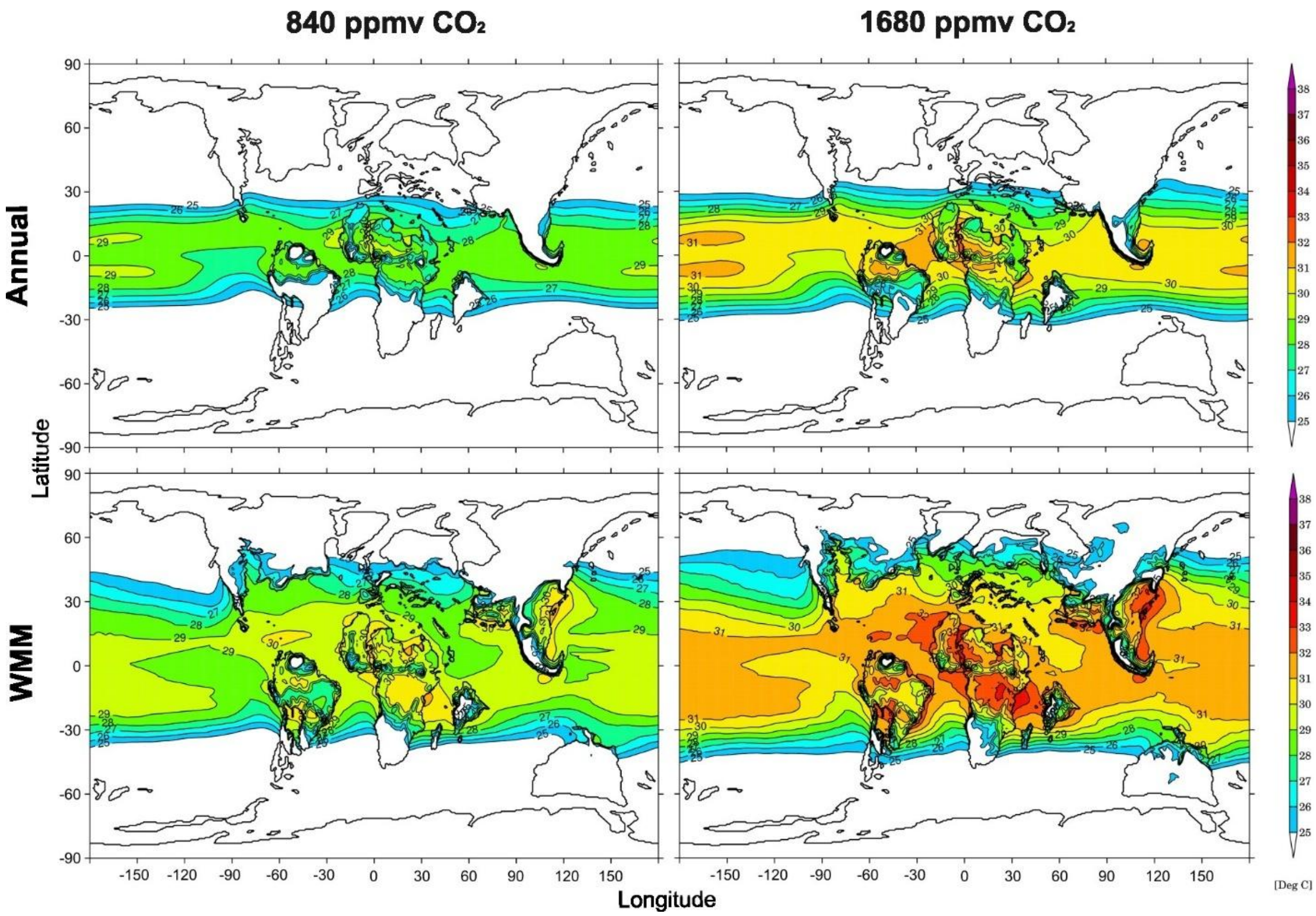

Fig. S7. **Heat stress metrics for the Maastrichtian.**
1.5m mean annual wet-bulb air temperatures (°C), and 1.5m Warm Month Mean (WMM) wet-bulb air temperatures (°C).

Other Supplementary Materials for this manuscript include the following:

Data S1. Raw data for clumped and phosphate oxygen isotope measurements

Data S2. FTIR indices

Data S3. GlobTherm dataset subset of homeothermic endotherms.

REFERENCES

1. N. C. Wu, F. Seebacher, Physiology can predict animal activity, exploration, and dispersal. *Commun. Biol.* **5**, 109 (2022).

2. J. R. Codd, P. L. Manning, M. A. Norell, S. F. Perry, Avian-like breathing mechanics in maniraptoran dinosaurs. *Proc. R. Soc. B* **275**, 157–161 (2008).

3. A. M. Bailleul, J. O'Connor, M. H. Schweitzer, Dinosaur paleohistology: Review, trends and new avenues of investigation. *PeerJ* **7**, e7764 (2019).

4. A. Clarke, Dinosaur energetics: Setting the bounds on feasible physiologies and ecologies. *Am. Nat.* **182**, 283–297 (2013).

5. A. A. Chiarenza, J. L. Cantalapiedra, L. A. Jones, S. Gamboa, S. Galván, A. J. Farnsworth, P. J. Valdes, G. Sotelo, S. Varela, Early Jurassic origin of avian endothermy and thermophysiological diversity in dinosaurs. *Curr. Biol.* **34**, 2517–2527 (2024).

6. A. A. Chiarenza, P. D. Mannion, A. Farnsworth, M. T. Carrano, S. Varela, Climatic constraints on the biogeographic history of Mesozoic dinosaurs. *Curr. Biol.* **32**, 570–585.e3 (2022).

7. R. Amiot, C. Lecuyer, E. Buffetaut, G. Escarguel, F. Fluteau, F. Martineau, Oxygen isotopes from biogenic apatites suggest widespread endothermy in Cretaceous dinosaurs. *Earth Planet. Sci. Lett.* **246**, 41–54 (2006).

8. H. C. Fricke, R. R. Rogers, Multiple taxon–multiple locality approach to providing oxygen isotope evidence for warm-blooded theropod dinosaurs. *Geology* **28**, 799 (2000).

9. R. R. Dawson, D. J. Field, P. M. Hull, D. K. Zelenitsky, F. Therrien, H. P. Affek, Eggshell geochemistry reveals ancestral metabolic thermoregulation in Dinosauria. *Sci. Adv.* **6**, eaax9361 (2020).

10. R. A. Eagle, T. Tütken, T. S. Martin, A. K. Tripati, H. C. Fricke, M. Connely, R. L. Cifelli, J. M. Eiler, Dinosaur body temperatures determined from isotopic (^{13}C-^{18}O) ordering in fossil biominerals. *Science* **333**, 443–445 (2011).

11. R. A. Eagle, M. Enriquez, G. Grellet-Tinner, A. Pérez-Huerta, D. Hu, T. Tütken, S. Montanari, S. J. Loyd, P. Ramirez, A. K. Tripati, M. J. Kohn, T. E. Cerling, L. M. Chiappe, J. M. Eiler, Isotopic ordering in eggshells reflects body temperatures and suggests differing thermophysiology in two Cretaceous dinosaurs. *Nat. Commun.* **6**, 8296 (2015).

12. A. H. Laskar, D. Mohabey, S. K. Bhattacharya, M.-C. Liang, Variable thermoregulation of Late Cretaceous dinosaurs inferred by clumped isotope analysis of fossilized eggshell carbonates. *Heliyon* **6**, e05265 (2020).

13. M. Tagliavento, S. Hofmann, M. E. Böttcher, M. Peltz, J. Fiebig, Clumped isotope analysis of (bio)apatite using a Kiel IV device in long-integration dual-inlet mode. *Rapid Commun. Mass Spectrom.* **37**, e9656 (2023).

14. J. Wiemann, I. Menéndez, J. M. Crawford, M. Fabbri, J. A. Gauthier, P. M. Hull, M. A. Norell, D. E. G. Briggs, Fossil biomolecules reveal an avian metabolism in the ancestral dinosaur. *Nature* **606**, 522–526 (2022).

15. C. R. Marshall, D. V. Latorre, C. J. Wilson, T. M. Frank, K. M. Magoulick, J. B. Zimmt, A. W. Poust, Absolute abundance and preservation rate of *Tyrannosaurus rex*. *Science* **372**, 284–287 (2021).

16. S. L. Brusatte, M. A. Norell, T. D. Carr, G. M. Erickson, J. R. Hutchinson, A. M. Balanoff, G. S. Bever, J. N. Choiniere, P. J. Makovicky, X. Xu, Tyrannosaur paleobiology: New research on ancient exemplar organisms. *Science* **329**, 1481–1485 (2010).

17. H. Pontzer, V. Allen, J. R. Hutchinson, Biomechanics of running indicates endothermy in bipedal dinosaurs. *PLOS ONE* **4**, e7783 (2009).

18. G. M. Erickson, Assessing dinosaur growth patterns: A microscopic revolution. *Trends Ecol. Evol.* **20**, 677–684 (2005).

19. R. E. Barrick, W. J. Showers, Thermophysiology of *Tyrannosaurus rex*: Evidence from oxygen isotopes. *Science* **265**, 222–224 (1994).

20. P. Ghosh, J. Adkins, H. Affek, B. Balta, W. Guo, E. A. Schauble, D. Schrag, J. M. Eiler, 13C–18O bonds in carbonate minerals: A new kind of paleothermometer. *Geochim. Cosmochim. Acta* **70**, 1439–1456 (2006).

21. M. D. Deak, W. P. Porter, P. D. Mathewson, D. M. Lovelace, R. J. Flores, A. K. Tripati, R. A. Eagle, D. M. Schwartz, M. T. Butcher, Metabolic skinflint or spendthrift? Insights into ground sloth integument and thermophysiology revealed by biophysical modeling and clumped isotope paleothermometry. *J. Mamm. Evol.* **32**, 1 (2025).

22. M. L. Griffiths, R. A. Eagle, S. L. Kim, R. J. Flores, M. A. Becker, H. M. Maisch, R. B. Trayler, R. L. Chan, J. McCormack, A. A. Akhtar, A. K. Tripati, K. Shimada, Endothermic physiology of extinct megatooth sharks. *Proc. Natl. Acad. Sci. U.S.A.* **120**, e2218153120 (2023).

23. N. Löffler, J. Fiebig, A. Mulch, T. Tütken, B. C. Schmidt, D. Bajnai, A. C. Conrad, U. Wacker, M. E. Böttcher, Refining the temperature dependence of the oxygen and clumped isotopic compositions of structurally bound carbonate in apatite. *Geochim. Cosmochim. Acta* **253**, 19–38 (2019).

24. T. D. Carr, A high-resolution growth series of *Tyrannosaurus rex* obtained from multiple lines of evidence. *PeerJ* **8**, e9192 (2020).

25. M. J. Kohn, T. E. Cerling, "12. Stable isotope compositions of biological apatite" in *Phosphates*, M. J. Kohn, J. Rakovan, J. M. Hughes, Eds. (De Gruyter, 2002), pp. 455–488.

26. H. C. Fricke, R. R. Rogers, R. Backlund, C. N. Dwyer, S. Echt, Preservation of primary stable isotope signals in dinosaur remains, and environmental gradients of the Late Cretaceous of Montana and Alberta. *Palaeogeogr. Palaeoclimatol. Palaeoecol.* **266**, 13–27 (2008).

27. T. M. Cullen, F. J. Longstaffe, U. G. Wortmann, L. Huang, F. Fanti, M. B. Goodwin, M. J. Ryan, D. C. Evans, Large-scale stable isotope characterization of a Late Cretaceous dinosaur-dominated ecosystem. *Geology* **48**, 546–551 (2020).

28. R. A. Eagle, E. A. Schauble, A. K. Tripati, T. Tütken, R. C. Hulbert, J. M. Eiler, Body temperatures of modern and extinct vertebrates from ^{13}C-^{18}O bond abundances in bioapatite. *Proc. Natl. Acad. Sci. U.S.A.* **107**, 10377–10382 (2010).

29. P. Iacumin, H. Bocherens, A. Mariotti, A. Longinelli, Oxygen isotope analyses of co-existing carbonate and phosphate in biogenic apatite: A way to monitor diagenetic alteration of bone phosphate? *Earth Planet. Sci. Lett.* **142**, 1–6 (1996).

30. A. Zazzo, C. Lécuyer, S. M. F. Sheppard, P. Grandjean, A. Mariotti, Diagenesis and the reconstruction of paleoenvironments: A method to restore original δ18O values of carbonate and phosphate from fossil tooth enamel. *Geochim. Cosmochim. Acta* **68**, 2245–2258 (2004).

31. A. Zazzo, C. Lécuyer, A. Mariotti, Experimentally-controlled carbon and oxygen isotope exchange between bioapatites and water under inorganic and microbially-mediated conditions. *Geochim. Cosmochim. Acta* **68**, 1–12 (2004).

32. M. Pellegrini, J. A. Lee-Thorp, R. E. Donahue, Exploring the variation of the δ18Op and δ18Oc relationship in enamel increments. *Palaeogeogr. Palaeoclimatol. Palaeoecol.* **310**, 71–83 (2011).

33. M. E. Karnes, R. L. Chan, J. P. Kuntz, M. L. Griffiths, K. Shimada, M. A. Becker, H. M. Maisch, R. A. Eagle, J. Brenner-Coltrain, S. Miller, S. L. Kim, Enigmatic carbonate isotope values in shark teeth: Evidence for environmental and dietary controls. *Palaeogeogr. Palaeoclimatol. Palaeoecol.* **635**, 111943 (2024).

34. A. Göhring, C. Von Carnap-Bornheim, V. Hilberg, C. Mayr, G. Grupe, Diet and species-specific oxygen isotope relationship and isotope spacing between structural carbonate and phosphate in archaeological mammalian bones. *Archaeol. Anthropol. Sci.* **11**, 2467–2487 (2019).

35. J. Botha, J. Lee–Thorp, M. Sponheimer, An examination of Triassic cynodont tooth enamel chemistry using Fourier transform infrared spectroscopy. *Calcif. Tissue Int.* **74**, 162–169 (2004).

36. C. M. Malanoski, A. Farnsworth, D. J. Lunt, P. J. Valdes, E. E. Saupe, Climate change is an important predictor of extinction risk on macroevolutionary timescales. *Science* **383**, 1130–1134 (2024).

37. T. S. Tobin, G. P. Wilson, J. M. Eiler, J. H. Hartman, Environmental change across a terrestrial Cretaceous-Paleogene boundary section in eastern Montana, USA, constrained by carbonate clumped isotope paleothermometry. *Geology* **42**, 351–354 (2014).

38. A. Clarke, P. Rothery, Scaling of body temperature in mammals and birds. *Funct. Ecol.* **22**, 58–67 (2008).

39. J. R. King, D. S. Farner, "Energy metabolism, thermoregulation and body temperature" in *Biology and Comparative Physiology of Birds* (Elsevier, 1961), pp. 215–288.

40. G. C. Grigg, F. Seebacherd, L. A. Beard, D. Morris, Thermal relations of large crocodiles, Crocodylus porosus, free—Ranging in a naturalistic situation. *Proc. Biol. Sci.* **265**, 1793–1799 (1998).

41. F. Seebacher, G. C. Grigg, Patterns of body temperature in wild freshwater crocodiles, Crocodylus johnstoni: Thermoregulation versus thermoconformity, seasonal acclimatization, and the effect of social interactions. *Copeia* **1997**, 549 (1997).

42. C. R. Johnson, G. J. W. Webb, C. Tanner, Thermoregulation in crocodilians—II. A telemetric study of body temperature in the Australian crocodiles, Crocodylus johnstoni and Crocodylus porosus. *Comp. Biochem. Physiol. A Physiol.* **53**, 143–146 (1976).

43. P. J. Markwick, Fossil crocodilians as indicators of Late Cretaceous and Cenozoic climates: Implications for using palaeontological data in reconstructing palaeoclimate. *Palaeogeogr. Palaeoclimatol. Palaeoecol.* **137**, 205–271 (1998).

44. J. F. Gillooly, A. P. Allen, E. L. Charnov, Dinosaur fossils predict body temperatures. *PLOS Biol.* **4**, e248 (2006).

45. E. M. Griebeler, Body temperatures in dinosaurs: What can growth curves tell us? *PLOS ONE* **8**, e74317 (2013).

46. M. J. Kohn, Predicting animal δ18O: Accounting for diet and physiological adaptation. *Geochim. Cosmochim. Acta* **60**, 4811–4829 (1996).

47. R. Amiot, C. Lécuyer, G. Escarguel, J.-P. Billon-Bruyat, E. Buffetaut, C. Langlois, S. Martin, F. Martineau, J.-M. Mazin, Oxygen isotope fractionation between crocodilian phosphate and water. *Palaeogeogr. Palaeoclimatol. Palaeoecol.* **243**, 412–420 (2007).

48. R. Amiot, D. Angst, S. Legendre, E. Buffetaut, F. Fourel, J. Adolfssen, A. André, A. V. Bojar, A. Canoville, A. Barral, J. Goedert, S. Halas, N. Kusuhashi, E. Pestchevitskaya, K. Rey, A. Royer, A. Á. F. Saraiva, B. Savary-Sismondini, J.-L. Siméon, A. Touzeau, Z. Zhou, C. Lécuyer, Oxygen isotope fractionation between bird bone phosphate and drinking water. *Sci. Nat.* **104**, 47 (2017).

49. B. N. Alvarez, G. A. Casal, L. M. Ibiricu, A. De Sosa Tomas, C. A. Suarez, Insights into paleoecology of the Bajo Barreal Formation (Upper Cretaceous), Patagonia, Argentina. *J. South Am. Earth Sci.* **116**, 103854 (2022).

50. N. Lazzerini, C. Lécuyer, R. Amiot, D. Angst, E. Buffetaut, F. Fourel, V. Daux, J. F. Betancort, J.-P. Flandrois, A. S. Marco, A. Lomoschitz, Oxygen isotope fractionation between bird eggshell calcite and body water: Application to fossil eggs from Lanzarote (Canary Islands). *Sci. Nat.* **103**, 81 (2016).

51. P. S. Druckenmiller, G. M. Erickson, D. Brinkman, C. M. Brown, J. J. Eberle, Nesting at extreme polar latitudes by non-avian dinosaurs. *Curr. Biol.* **31**, 3469–3478.e5 (2021).

52. J. M. Bennett, P. Calosi, S. Clusella-Trullas, B. Martínez, J. Sunday, A. C. Algar, M. B. Araújo, B. A. Hawkins, S. Keith, I. Kühn, C. Rahbek, L. Rodríguez, A. Singer, F. Villalobos,

M. Ángel Olalla-Tárraga, I. Morales-Castilla, GlobTherm, a global database on thermal tolerances for aquatic and terrestrial organisms. *Sci. Data* **5**, 180022 (2018).

53. A. Farnsworth, Y. T. E. Lo, P. J. Valdes, J. R. Buzan, B. J. W. Mills, A. S. Merdith, C. R. Scotese, H. R. Wakeford, Climate extremes likely to drive land mammal extinction during next supercontinent assembly. *Nat. Geosci.* **16**, 901–908 (2023).

54. A. A. Chiarenza, P. D. Mannion, D. J. Lunt, A. Farnsworth, L. A. Jones, S.-J. Kelland, P. A. Allison, Ecological niche modelling does not support climatically-driven dinosaur diversity decline before the Cretaceous/Paleogene mass extinction. *Nat. Commun.* **10**, 1091 (2019).

55. A. R. Fiorillo, R. S. Tykoski, A diminutive new tyrannosaur from the top of the world. *PLOS ONE* **9**, e91287 (2014).

56. D. A. Lawson, *Tyrannosaurus* and *Torosaurus*, Maestrichtian dinosaurs from Trans-Pecos, Texas. *J. Paleontol.* **50**, 158–164 (1976).

57. J. T. Voris, D. K. Zelenitsky, Y. Kobayashi, S. P. Modesto, F. Therrien, H. Tsutsumi, T. Chinzorig, K. Tsogtbaatar, A new Mongolian tyrannosauroid and the evolution of Eutyrannosauria. *Nature* **642**, 973–979 (2025).

58. S. M. Bernasconi, M. Daëron, K. D. Bergmann, M. Bonifacie, A. N. Meckler, H. P. Affek, N. Anderson, D. Bajnai, E. Barkan, E. Beverly, D. Blamart, L. Burgener, D. Calmels, C. Chaduteau, M. Clog, B. Davidheiser-Kroll, A. Davies, F. Dux, J. Eiler, B. Elliott, A. C. Fetrow, J. Fiebig, S. Goldberg, M. Hermoso, K. W. Huntington, E. Hyland, M. Ingalls, M. Jaggi, C. M. John, A. B. Jost, S. Katz, J. Kelson, T. Kluge, I. J. Kocken, A. Laskar, T. J. Leutert, D. Liang, J. Lucarelli, T. J. Mackey, X. Mangenot, N. Meinicke, S. E. Modestou, I. A. Müller, S. Murray, A. Neary, N. Packard, B. H. Passey, E. Pelletier, S. Petersen, A. Piasecki, A. Schauer, K. E. Snell, P. K. Swart, A. Tripati, D. Upadhyay, T. Vennemann, I. Winkelstern, D. Yarian, N. Yoshida, N. Zhang, M. Ziegler, InterCarb: A community effort to improve interlaboratory standardization of the carbonate clumped isotope thermometer using carbonate standards. *Geochem. Geophys. Geosyst.* **22**, e2020GC009588 (2021).

59. C. M. John, D. Bowen, Community software for challenging isotope analysis: First applications of 'Easotope' to clumped isotopes. *Rapid Commun. Mass Spectrom.* **30**, 2285–2300 (2016).

60. N. T. Anderson, J. R. Kelson, S. Kele, M. Daëron, M. Bonifacie, J. Horita, T. J. Mackey, C. M. John, T. Kluge, P. Petschnig, A. B. Jost, K. W. Huntington, S. M. Bernasconi, K. D. Bergmann, A unified clumped isotope thermometer calibration (0.5–1,100°C) using carbonate-based standardization. *Geophys. Res. Lett.* **48**, e2020GL092069 (2021).

61. N. T. Anderson, M. Bonifacie, A. B. Jost, J. Siebert, T. Bontognali, J. Horita, I. A. Müller, S. M. Bernasconi, K. D. Bergmann, Re-assessing the need for apatite- and dolomite-specific calibrations of the carbonate clumped isotope thermometer. *Geochem. Geophys. Geosyst.* **25**, e2023GC011049 (2024).

62. G. Larocca Conte, L. E. Lopes, A. H. Mine, R. B. Trayler, S. L. Kim, SPORA, a new silver phosphate precipitation protocol for oxygen isotope analysis of small, organic-rich bioapatite samples. *Chem. Geol.* **651**, 122000 (2024).

63. A. H. Mine, A. Waldeck, G. Olack, M. E. Hoerner, S. Alex, A. S. Colman, Microprecipitation and $\delta^{18}O$ analysis of phosphate for paleoclimate and biogeochemistry research. *Chem. Geol.* **460**, 1–14 (2017).

64. C. A. Chenery, V. Pashley, A. L. Lamb, H. J. Sloane, J. A. Evans, The oxygen isotope relationship between the phosphate and structural carbonate fractions of human bioapatite. *Rapid Commun. Mass Spectrom.* **26**, 309–319 (2012).

65. R. B. Trayler, P. V. Landa, S. L. Kim, Evaluating the efficacy of collagen isolation using stable isotope analysis and infrared spectroscopy. *J. Archaeol. Sci.* **151**, 105727 (2023).

66. P. J. Valdes, E. Armstrong, M. P. S. Badger, C. D. Bradshaw, F. Bragg, M. Crucifix, T. Davies-Barnard, J. J. Day, A. Farnsworth, C. Gordon, P. O. Hopcroft, A. T. Kennedy, N. S. Lord, D. J. Lunt, A. Marzocchi, L. M. Parry, V. Pope, W. H. G. Roberts, E. J. Stone, G. J. L. Tourte, J. H. T. Williams, The BRIDGE HadCM3 family of climate models: HadCM3@Bristol v1.0. *Geosci. Model Dev.* **10**, 3715–3743 (2017).

67. P. M. Cox, R. A. Betts, C. B. Bunton, R. L. H. Essery, P. R. Rowntree, J. Smith, The impact of new land surface physics on the GCM simulation of climate and climate sensitivity. *Climate Dynam.* **15**, 183–203 (1999).

68. N. Sagoo, P. Valdes, R. Flecker, L. J. Gregoire, The Early Eocene equable climate problem: Can perturbations of climate model parameters identify possible solutions? *Philos. Trans. A Math. Phys. Eng. Sci.* **371**, 20130123 (2013).

69. J. T. Kiehl, C. A. Shields, Sensitivity of the Palaeocene–Eocene Thermal Maximum climate to cloud properties. *Philos. Trans. A Math. Phys. Eng. Sci.* **371**, 20130093 (2013).

70. I. S. Fenton, T. Aze, A. Farnsworth, P. Valdes, E. E. Saupe, Origination of the modern-style diversity gradient 15 million years ago. *Nature* **614**, 708–712 (2023).

71. M. D. Cox, K. Bryan, A numerical model of the ventilated thermocline. *J. Phys. Oceanogr.* **14**, 674–687 (1984).

72. W. D. Collins, Parameterization of generalized cloud overlap for radiative calculations in general circulation models. *J. Atmos. Sci.* **58**, 3224–3242 (2001).

73. A. Farnsworth, D. J. Lunt, C. L. O'Brien, G. L. Foster, G. N. Inglis, P. Markwick, R. D. Pancost, S. A. Robinson, Climate sensitivity on geological timescales controlled by nonlinear feedbacks and ocean circulation. *Geophys. Res. Lett.* **46**, 9880–9889 (2019).

74. A. Farnsworth, D. J. Lunt, S. A. Robinson, P. J. Valdes, W. H. G. Roberts, P. D. Clift, P. Markwick, T. Su, N. Wrobel, F. Bragg, S.-J. Kelland, R. D. Pancost, Past East Asian monsoon evolution controlled by paleogeography, not CO_2. *Sci. Adv.* **5**, eaax1697 (2019).

75. C. D. Jones, "Numerical modeling of the global climate and carbon cycle system" in *Climate Change* (Elsevier, 2021), pp. 67–91.

76. J. W. B. Rae, Y. G. Zhang, X. Liu, G. L. Foster, H. M. Stoll, R. D. M. Whiteford, Atmospheric CO_2 over the past 66 million years from marine archives. *Annu. Rev. Earth Planet. Sci.* **49**, 609–641 (2021).

77. D. O. Gough, Solar interior structure and luminosity variations. *Sol. Phys.* **74**, 21–34 (1981).

78. S. C. Sherwood, M. Huber, An adaptability limit to climate change due to heat stress. *Proc. Natl. Acad. Sci. U.S.A.* **107**, 9552–9555 (2010).

79. R. Davies-Jones, An efficient and accurate method for computing the wet-bulb temperature along pseudoadiabats. *Mon. Weather Rev.* **136**, 2764–2785 (2008).

80. D. Bolton, The computation of equivalent potential temperature. *Mon. Weather Rev.* **108**, 1046–1053 (1980).

81. R. Stull, Wet-bulb temperature from relative humidity and air temperature. *J. Appl. Meteorol. Climatol.* **50**, 2267–2269 (2011).

82. J. R. Buzan, K. Oleson, M. Huber, Implementation and comparison of a suite of heat stress metrics within the Community Land Model version 4.5. *Geosci. Model Dev.* **8**, 151–170 (2015).

83. S. Asseng, D. Spänkuch, I. M. Hernandez-Ochoa, J. Laporta, The upper temperature thresholds of life. *Lancet Planet. Heath* **5**, e378–e385 (2021).

84. B. Leroy, C. N. Meynard, C. Bellard, F. Courchamp, virtualspecies, an R package to generate virtual species distributions. *Ecography* **39**, 599–607 (2016).

85. C. R. Scotese, PALEOMAP PaleoAtlas for GPlates and the PaleoData Plotter program. *Geol. Soc. Am.* , (2016).

86..T Kocsis, C. R. Scotese, Mapping paleocoastlines and continental flooding during the Phanerozoic. *Earth Sci. Rev.* **213**, 103463 (2021).

87. A. D. Miall, *The Sedimentary Basins of the United States and Canada* (Elsevier Science, ed. 1, 2008).

88. J. S. Slattery, W. A. Cobban, K. C. Mckinney, P. J. Harries, A. L. Sandness, paper presented at the Wyoming Geological Association 68th Annual Field Conference, Casper, WY, June 2013.

89. R. Fernandes, M. Sponheimer, P. Roberts, TOOTHFIR: Presenting a dataset and a preliminary meta-analysis of Fourier Transform Infra-red Spectroscopy indices from archaeological and palaeontological tooth enamel. *Quat. Int.* **650**, 77–85 (2023).

90. R. J. Hijmans, terra: Spatial Data Analysis (2020); https://doi.org/10.32614/CRAN.package.terra.

91. L. A. Jones, C. D. Dean, B. J. Allen, H. B. Drage, J. T. Flannery-Sutherland, W. Gearty, A. A. Chiarenza, E. M. Dillon, B. M. Farina, P. L. Godoy, Ten simple rules to follow when cleaning occurrence data in palaeobiology. *Palaeontology* **68**, e70028 (2025).

92. L. A. Jones, W. Gearty, B. J. Allen, K. Eichenseer, C. D. Dean, S. Galván, M. Kouvari, P. L. Godoy, C. S. C. Nicholl, L. Buffan, E. M. Dillon, J. T. Flannery-Sutherland, A. A. Chiarenza, palaeoverse: A community-driven R package to support palaeobiological analysis. *Methods Ecol. Evol.* **14**, 2205–2215 (2023).

93. D. W. Fowler, Revised geochronology, correlation, and dinosaur stratigraphic ranges of the Santonian-Maastrichtian (Late Cretaceous) formations of the Western Interior of North America. *PLOS ONE* **12**, e0188426 (2017).

94. D. L. Lofgren, *The Bug Creek Problem and the Cretaceous-Tertiary Transition at McGuire Creek, Montana* (University of California Press, 1995).

95. C. Martin, I. Bentaleb, R. Kaandorp, P. Iacumin, K. Chatri, Intra-tooth study of modern rhinoceros enamel δ18O: Is the difference between phosphate and carbonate δ18O a sound diagenetic test? *Palaeogeogr. Palaeoclimatol. Palaeoecol.* **266**, 183–189 (2008).

96. D. L. Dettman, K. C. Lohmann, Oxygen isotope evidence for high-altitude snow in the Laramide Rocky Mountains of North America during the Late Cretaceous and Paleogene. *Geology* **28**, 243 (2000).

97. M. M. Beasley, E. J. Bartelink, L. Taylor, R. M. Miller, Comparison of transmission FTIR, ATR, and DRIFT spectra: Implications for assessment of bone bioapatite diagenesis. *J. Archaeol. Sci.* **46**, 16–22 (2014).

98. D. Roche, L. Ségalen, E. Balan, S. Delattre, Preservation assessment of Miocene–Pliocene tooth enamel from Tugen Hills (Kenyan Rift Valley) through FTIR, chemical and stable-isotope analyses. *J. Archaeol. Sci.* **37**, 1690–1699 (2010).

99. M. Sponheimer, J. A. Lee-Thorp, Alteration of enamel carbonate environments during fossilization. *J. Archaeol. Sci.* **26**, 143–150 (1999).

100. K. Rey, R. Amiot, F. Fourel, T. Rigaudier, F. Abdala, M. O. Day, V. Fernandez, F. Fluteau, C. France-Lanord, B. S. Rubidge, R. M. Smith, P. A. Viglietti, B. Zipfel, C. Lécuyer, Global climate perturbations during the Permo-Triassic mass extinctions recorded by continental tetrapods from South Africa. *Gondwana Res.* **37**, 384–396 (2016).
